# A direct interaction of JAM-C with the tight junction scaffold protein ZO-2

**DOI:** 10.1038/s41598-026-56546-x

**Published:** 2026-06-10

**Authors:** Annika Schulte, Mariel F. Schwietzer, Frauke Brinkmann, Valentin Teuber, Sandra Citi, Mikio Furuse, Michel Aurrand-Lions, Klaus Ebnet

**Affiliations:** 1 Institute-associated Research Group Cell Adhesion and Cell Polarity, Institute of Medical Biochemistry, ZMBE, University of Münster, Von- Esmarch-Str. 56, D-48149 Münster, Germany; 2 Department of Molecular and Cellular Biology, University of Geneva, Geneva, 1205 Switzerland; 3 Division of Cell Structure, National Institute for Physiological Sciences, National Institute of Natural Sciences, Okazaki, Aichi Japan; 4 Institut Paoli-Calmettes, CRCM, Equipe Labellisée Ligue 2020, Team Leuko/stromalinteractions in normal and pathological haematopoiesis, Aix Marseille University, CNRS, INSERM, Marseille France; 5 Cells-in-Motion Cluster of Excellence (EXC 1003-CiM), University of Münster, Münster, Germany

**Keywords:** JAM-A, JAM-C, PDZ domain, Tight junction, ZO-2, Biochemistry, Biophysics, Cell biology

## Abstract

Tight junctions are sites of cell-cell contacts at the apical region of epithelial junctions that are involved in barrier formation, cellular signaling, and cell-cell adhesion. Tight junctions are formed by integral membrane proteins associated with cytoplasmic scaffolding and adapter proteins through which they are linked to the underlying actomyosin and microtubule cytoskeletons. Here, we have addressed the interaction of the Junctional Adhesion Molecule (JAM)-C with the zonula adherens (ZO) protein ZO-2. Using a combination of cell-based recruitment assays and biochemical in vitro experiments, we find that JAM-C and ZO-2 directly interact in a PDZ domain-dependent manner. Notably, the interaction requires PDZ domain 3 as well as the SH3 domain of ZO-2, indicating that ZO-2 forms a functional supramodule to interact with JAM-C. We also found that JAM-C is specifically localized to tight junctions in polarized epithelial cells and that JAM-A suppresses JAM-C mRNA expression in these cells. Our findings have implications for important aspects of tight junction biology, including mechanosensing and liquid–liquid phase separation.

**Supplementary Information:**

The online version contains supplementary material available at 10.1038/s41598-026-56546-x.

## Introduction

Tight junctions (TJ) are sites of direct contacts between adjacent epithelial cells at the apical region of intercellular junctions^1^. The direct cell-cell contact sites are established by claudins, a family of integral membrane proteins with four membrane-spanning domains^2^, which interact in cis and trans to form a mesh-like network of strands which prevents an uncontrolled passage of solutes and water along the paracellular pathway^3^. Additional integral membrane proteins at the TJs include members of the TJ-associated Marvel protein (TAMP) family including occludin, tricellulin and MarvelD3, as well as members of the immunoglobulin superfamily (IgSF), such as the Coxsackie and adenovirus receptor (CAR) and the junctional adhesion molecules (JAMs) JAM-A, JAM-C and JAM4 ^4^.

All integral membrane proteins localized at the TJs interact through their cytoplasmic tails with peripheral membrane proteins which serve as scaffolds and adaptors^5^. These proteins are characterized by various protein-protein interaction domains including PDZ domains, Src homology 3 (SH3) domains, guanylate kinase-like (GuK) domains, or proline-rich (PR) domains. The presence of multiple protein-protein interaction domains is the basis of the principal function of scaffolding proteins, i.e. the assembly of large protein complexes for intermolecular cross-talk, their localization at specific submembranous sites, and their functional interaction with the microtubule network and the actomyosin cytoskeleton^5^.

A family of scaffolding proteins that is particularly highly enriched at the TJs of epithelial cells and the postsynaptic density of neuronal cells is the membrane-associated guanylate kinase (MAGUK) family^6,7^. Members of this family are characterized by the presence of one or several PDZ domains, an SH3 domain, and a catalytically inactive GuK domain. Importantly, one of the PDZ domains often exists in a tandem organization with the SH3 and the GuK domains, building up the PDZ–SH3–GuK (PSG) core unit, a supramodule with distinct binding properties with regard to the isolated domains^8^. MAGUK family members located at the TJs include the zonula occludens (ZO) proteins ZO-1, ZO-2 and ZO-3^9^, Protein associated with Lin-7 1 (Pals1)^10^, and Membrane-associated guanylate kinase inverted 1 (MAGI-1)^11^. Other scaffolding proteins at the TJs include the partitioning defective (PAR) polarity proteins PAR-3 and PAR-6, and two proteins with 10 or 13 PDZ domains, Pals1-associated tight junction protein (PATJ) and Multiple PDZ domain protein 1 (MUPP1), respectively^5^. Most likely, additional scaffolding proteins localize either stably or transiently to TJs^12^ to regulate the TJ dynamics and plasticity^13^. Examples for the role of scaffolding proteins in TJ plasticity include their ability to sense and transform mechanical forces into cellular responses^14–17^, and their ability to self-organize into biomolecular condensates in a process called liquid–liquid phase separation (LLPS)^18,19^. Importantly, these functions depend on their association with cell-cell adhesion receptors including claudins or JAM-A^15,20–23^.

JAM-C is a member of the JAM family of cell-cell adhesion receptors^24^. It is expressed by a large number of different cell types including epithelial and endothelial cells, various leukocyte subsets, cells of the nervous system, and cells of the reproductive system^25,26^. Studies with JAM-C knockout mice revealed important roles in the myelination of peripheral nerves^27^, in the differentiation of male germ cells^28^, in hematopoiesis^29^, and during inflammatory responses^30^. Its role in epithelial cells is less well understood. It localizes to TJs in various cultured cell lines of epithelial origin^31–33^ and of primary retinal pigment epithelial (RPE) cells^34,35^. In addition, JAM-C interacts with the TJ scaffolding proteins ZO-1 and Par-3^36^.

The important roles of the interaction of scaffolding proteins with cell-cell adhesion receptors at the TJs prompted us to address a putative interaction of JAM-C with the scaffolding protein ZO-2. Using a heterologous CHO cell system, we find that JAM-C is sufficient to recruit ZO-2 to cell-cell contacts. The recruitment of ZO-2 is mediated through the C-terminal PDZ binding motif (PBM) of JAM-C. Recruitment assays and biochemical experiments indicate that the interaction is direct and requires both PDZ domain 3 and the SH3 domain of ZO-2. We identify two hydrophobic amino acid residues in the ZO-2 SH3 domain, that are predicted to interact with the PBM of JAM-C. We also find that JAM-C recruits the adapter protein cingulin (CGN) to cell-cell contacts. Finally, we find that JAM-C expression at cell-cell junctions of polarized epithelial cells is upregulated in the absence of JAM-A through a mechanism that involves increased mRNA expression. Our findings indicate that JAM-C directly interacts with ZO-2 through the PSG core module, and that the ZO-2 core module forms a functional supramodule. Our findings also indicate that JAM-C can associate with several scaffolding and adapter proteins at TJs including ZO-1, ZO-2 and CGN, and that it preferentially interacts with ZO-2 over ZO-1. Our findings have implications for various aspects of TJ biology including mechanosensation and LLPS.

## Results

### JAM-C promotes the recruitment of ZO-2 to cell-cell contacts

Given the important role of cell adhesion receptors and their associated scaffolding proteins in mechanosensing and phase separation, we addressed the question of whether JAM-C contributes to the localization of ZO-2 at cell-cell contacts. We used CHO cells since these cells lack expression of classical adhesion receptors including JAMs, cadherins or nectins. Scaffolding proteins that are normally recruited by cell adhesion receptors reside in the cytoplasm and are absent at cell-cell contacts. Importantly, ectopic expression of these cell adhesion receptors results in the recruitment of their cytoplasmic binding partners to cell-cell junctions. For example, ZO-1 and Par-3 are recruited by JAM-A, JAM-C and VE-cadherin^36–38^, α-catenin, β-catenin and p120^ctn^ are recruited by E-cadherin and VE-cadherin^38,39^, and Par-3 is recruited by VE-cadherin and Necl-4^38,40^. CHO cells are thus a suitable system to analyze the ability of a single adhesion receptor to recruit a specific scaffolding protein to intercellular junctions.

**Fig. 1:**
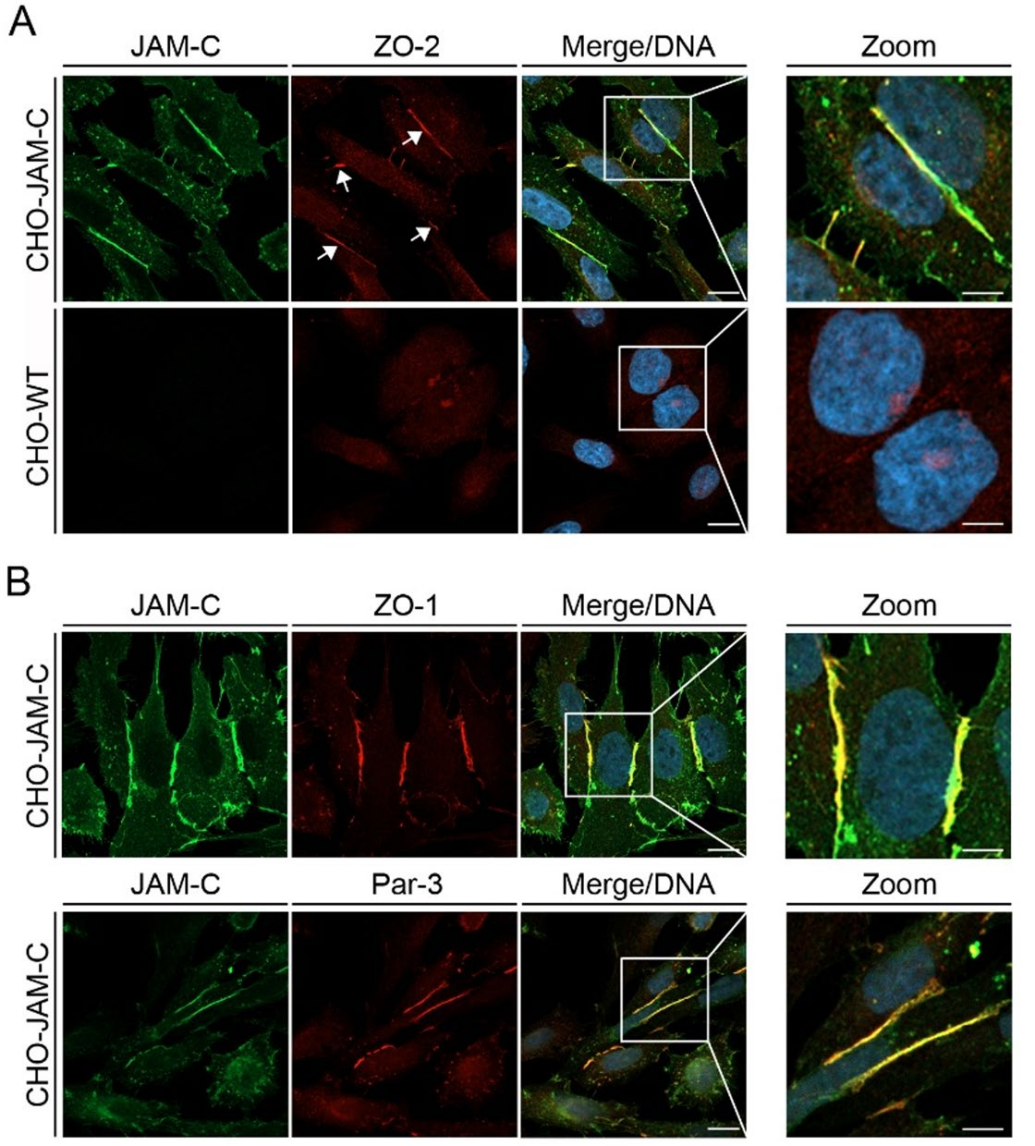
ZO-2 is recruited to JAM-C-based cell-cell contacts in CHO cells. (**A**) CHO WT cells (CHO-WT) or CHO cells stably expressing mJAM-C (CHO-JAM-C) were stained with antibodies against JAM-C together with antibodies against ZO-2 as indicated. Arrows indicate ZO-2-positive cell-cell contacts. (**B**) CHO-JAM-C cells were stained with antibodies against JAM-C together with antibodies against ZO-1 or Par-3 as indicated. Right panels in (A) and (B) show magnifications of the insets marked by white squares in the Merge/DAPI images. Data are representative for *N* = 4 independent experiments. Scale bars: 10 μm (regular images), 5 μm (zoomed insets).

Ectopic expression of JAM-C in these cells resulted in a robust recruitment of endogenous ZO-2 to JAM-C-based cell-cell contacts (Fig. 1A). The recruitment of ZO-2 was comparable to that of ZO-1 and Par-3, two scaffolding proteins previously shown to interact with JAM-C^36^ (Fig. 1B).

**Fig. 2:**
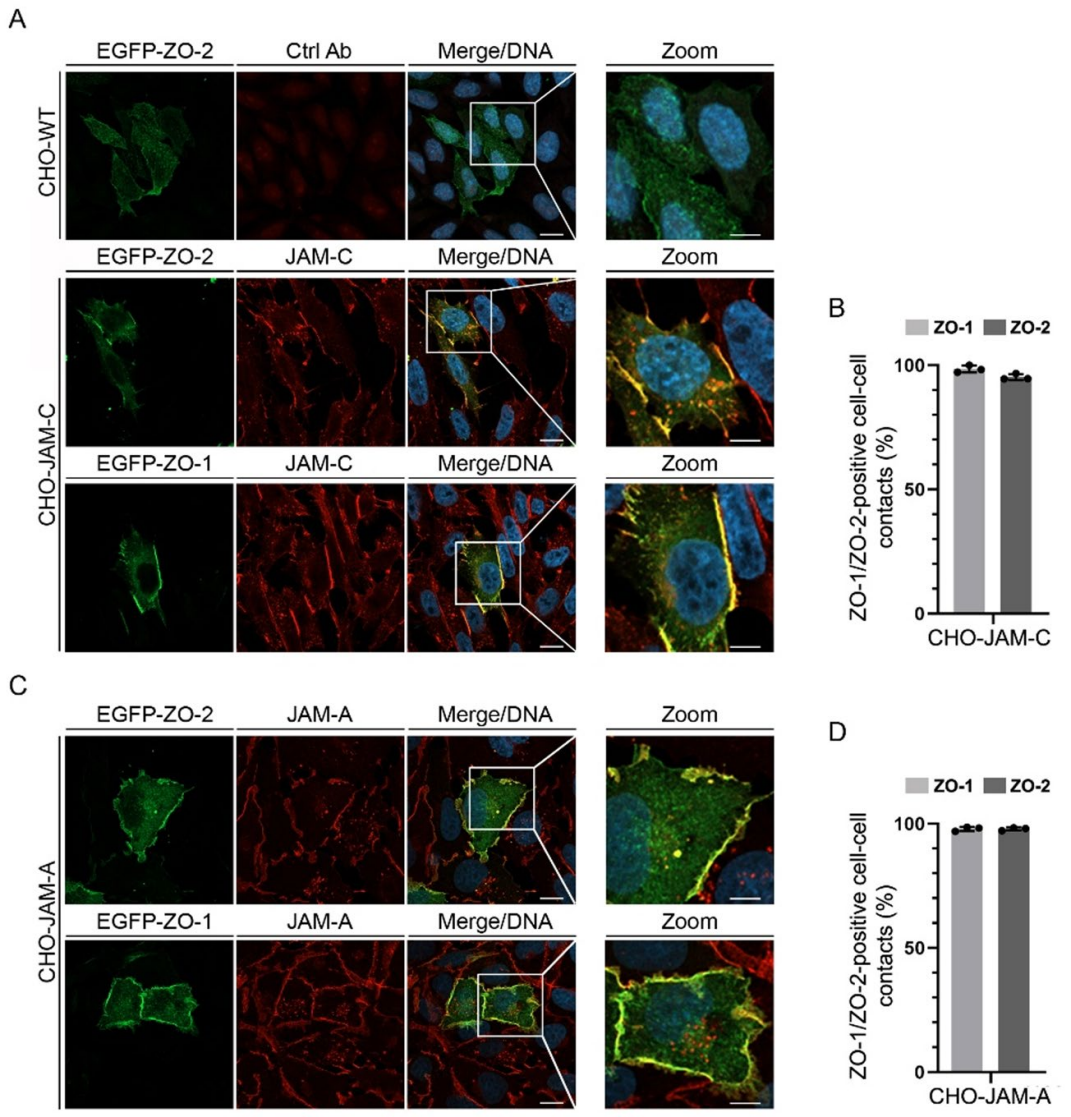
JAM-C recruits ZO-2 to cell-cell contacts as efficiently as ZO-1. (**A**) CHO WT cells (CHO-WT) or CHO cells stably expressing mJAM-C (CHO-JAM-C) were transfected with EGFP-ZO-2 or EGFP-ZO-1 as indicated and stained with antibodies against JAM-C. Right panels (Zoom) show magnifications of the insets marked by white squares in the Merge/DAPI images. Scale bars: 10 μm (regular images), 5 μm (zoomed insets). (**B**) Statistical evaluation of ZO-1 and ZO-2 recruitment by JAM-C. The bar graph shows the fraction of EGFP-ZO-1- or EGFP-ZO-2-positive cell-cell contacts in CHO-JAM-C cells. (**C**) CHO-JAM-A cells were transfected with EGFP-ZO-2 or EGFP-ZO-1 as indicated and stained with antibodies against JAM-A. Right panels (Zoom) show magnifications of the insets marked by white squares in the Merge/DAPI images. Scale bars: 10 μm (regular images), 5 μm (zoomed insets). (**D**) Statistical evaluation of ZO-1 and ZO-2 recruitment by JAM-A. The bar graph shows the fraction of EGFP-ZO-1- or EGFP-ZO-2-positive cell-cell contacts in CHO-JAM-A cells. In panels B and D, data are derived from *N* = 3 independent experiments, each data point represents the mean value of one experiment. Statistical analysis was performed with unpaired Student’s t-test. Data are presented as mean values ± SD. Number of analyzed cell-cell contacts: panel B (CHO-JAM-C): n(JAM-C–ZO-1): 195; n(JAM-C–ZO-2): 182; panel D (CHO-JAM-A): n(JAM-A–ZO-1): 196; n(JAM-A–ZO-2): 187.

Similar to endogenous ZO-2, EGFP-ZO-2 was absent at cell-cell contacts of wild-type CHO (CHO-WT) cells but was prominently localized at cell-cell contacts of CHO-JAM-C cells (Fig. 2A). The recruitment of EGFP-ZO-2 by JAM-C was similarly efficient as the recruitment of EGFP-ZO-1 (Fig. 2B), which has been described to be recruited by JAM-A in CHO cells^37^. These observations indicated that JAM-C can efficiently recruit ZO-2 to cell-cell contacts. To compare this function of JAM-C with that of JAM-A, which has previously been described to interact with ZO-1 and ZO-2^41–43^, we performed analogous experiments with JAM-A-transfected CHO cells. Both ZO proteins were localized at cell-cell contacts in CHO-JAM-A cells with similar frequencies as in CHO-JAM-C cells (Fig. 2C, D), indicating that JAM-C and JAM-A are equally potent in recruiting the two ZO proteins to cell-cell contacts in CHO cells.

## JAM-C interacts with ZO-2 through its PDZ domain binding motif

**Fig. 3:**
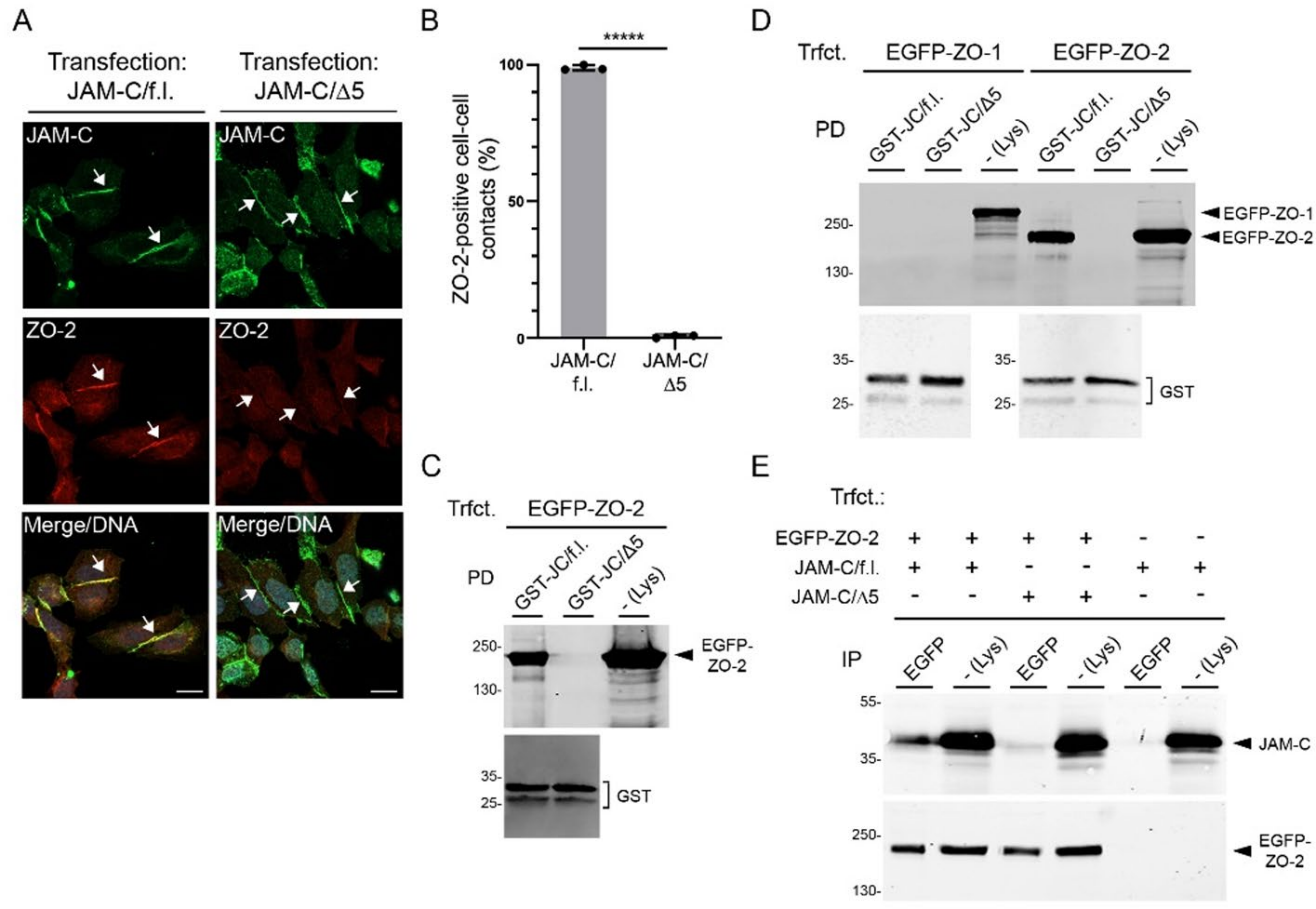
JAM-C interacts with ZO-2 through a PDZ domain-mediated interaction. (**A**) CHO WT cells were transiently transfected with JAM-C full length (JAM-C/f.l.) or a JAM-C truncation mutant lacking the PDZ domain binding motif (JAM-C/Δ5). Cells were stained with antibodies against JAM-C and ZO-2. Scale bars: 10 μm. (**B**) Statistical analysis of ZO-2 recruitment by JAM-C/f.l. and JAM-C/Δ5. The bar graph shows the fraction of cell-cell contacts in which endogenous ZO-2 co-localizes with either JAM-C/f.l. or JAM-C/Δ5. Statistical analysis was performed with unpaired Student’s t-test. Data are derived from *N* = 3 independent experiments, each data point represents the mean value of one experiment. Data are presented as mean values ± SD. Number of analyzed cell-cell contacts: n(JAM-C/f.l.): 360; n(JAM-C/Δ5): 317. *****P < 0.00001. (**C**) GST-pulldown experiment. GST fusion proteins containing the entire cytoplasmic domain of JAM-C (GST-JC/f.l.) or a cytoplasmic domain deletion mutant lacking the PBM (GST-JC/Δ5) were incubated with lysates from HEK293T cells transfected with EGFP-tagged full length ZO-2 (EGFP-ZO-2). Bound proteins were analyzed by immunoblotting using anti-GFP antibodies. The lane labeled “- (Lys)” contains a postnuclear supernatant of transfected cells (25% of input). The amounts of GST fusion proteins used in the pulldown experiments were analyzed with anti-GST antibodies (bottom panel, 10% of input). Data are representative of *N* = 3 independent experiments. (**D**) GST-pulldown experiment. GST fusion proteins were incubated with lysates from EGFP-ZO-1- or EGFP-ZO-2-transfected HEK293T cells and analyzed as described for panel C. Data are representative of *N* = 3 independent experiments. (**E**) Co-immunoprecipitation experiment. HEK293T cells were transfected with EGFP-ZO-2 together with either JAM-C/f.l. or JAM-C/∆5 as indicated. Cells transfected with JAM-C alone (lanes 5 and 6) served as IP control to test for unspecific precipitation of JAM-C. Immunoprecipitates were blotted with antibodies against JAM-C (top panels, 90% of input) or with antibodies against EGFP (bottom panels, 10% of input). Lanes labeled “- (Lys)” contain postnuclear supernatants of transfected cells (1.25% of input). Data are representative of *N* = 3 independent experiments. Abbreviations: Trfct., transfection; IP, immunoprecipitation.

The interaction of JAM-C with the TJ scaffold proteins ZO-1 and Par-3 is mediated by its C-terminal PBM (-SSFVI_COOH_)^36^. To test if the recruitment of ZO-2 involves the PBM of JAM-C, we expressed full length JAM-C (JAM-C/f.l.) or a PBM deletion mutant of JAM-C (JAM-C/∆5) in CHO-WT cells. Endogenous ZO-2 was efficiently recruited by JAM-C/f.l. but not by JAM-C/∆5 (Fig. 3A, B), strongly suggesting that JAM-C recruits ZO-2 through one (or several) of the three ZO-2 PDZ domains. As further support of this notion, pulldown experiments showed that EGFP-ZO-2 could be efficiently precipitated from HEK293T cell lysates with a GST fusion protein containing the intact cytoplasmic domain of JAM-C (GST-JAM-C/f.l.) but not with a GST-JAM-C cytoplasmic domain fusion protein that lacks the PBM (GST-JAM-C/∆5) (Fig. 3C). Interestingly, the GST-JAM-C fusion protein precipitated ZO-2 but not ZO-1 from lysates of transfected HEK293T cells (Fig. 3D), suggesting a strong preference of JAM-C for ZO-2 over ZO-1, as previously demonstrated for JAM-A^43^.

To further address the interaction of JAM-C and ZO-2 in cells we performed co-immunoprecipitation (CoIP) experiments from transfected HEK293T cells. ZO-2 immunoprecipitates contained JAM-C/f.l. but not JAM-C/∆5 (Fig. 3E). Together, these observations strongly suggest that JAM-C and ZO-2 interact in a PDZ domain-dependent manner and that JAM-C interacts preferentially with ZO-2 over ZO-1.

## JAM-C interacts with ZO-2 through the PDZ-SH3-GuK core module

**Fig. 4:**
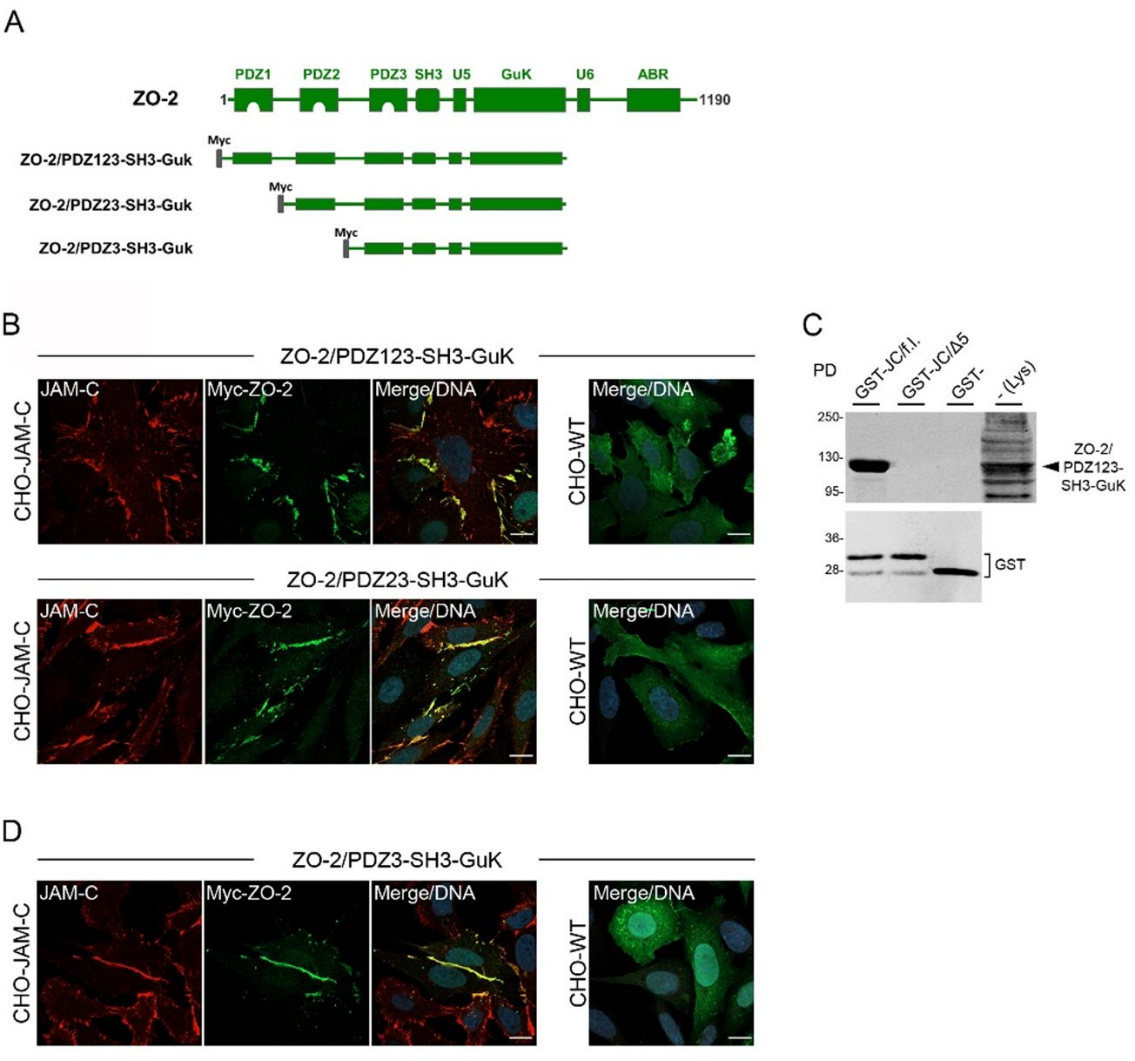
JAM-C interacts with a ZO-2 construct containing the PDZ,SH3 and GuK domains of ZO-2. (**A**) Cartoon of ZO-2 deletion constructs. (**B**) CHO-JAM-C or CHO-WT cells were transfected with the ZO-2/PDZ123-SH3-GuK construct (top panel) or with the ZO-2/PDZ23-SH3-GuK construct (bottom panel) and stained with antibodies against JAM-C (red) and against the Myc tag (green). Note that both constructs are enriched at JAM-C-positive cell junctions in CHO-JAM-C cells but are localized diffusely in the cytoplasm of CHO-WT cells. Data are representative of *N* = 3 independent experiments. Scale bars: 10 μm. (**C**) GST-pulldown experiment. Top panel: GST fusion proteins (GST-JC/f.l., GST-JC/Δ5, GST-) were incubated with lysates from HEK293T cells transfected with the Myc-tagged ZO-2/PDZ123-SH3-GuK construct. Bound proteins were analyzed by immunoblotting using anti-Myc antibodies. The lane labeled “- (Lys)” contains a postnuclear supernatant of transfected cells (25% of input). Bottom panel: The amounts of GST fusion proteins used in the pulldown experiments were analyzed with anti-GST antibodies (10% of input). Data are representative of *N* = 3 independent experiments. (**D**) CHO-JAM-C or CHO-WT cells were transfected with a ZO-2 construct lacking PDZ1 and PDZ2 (ZO-2/PDZ3-SH3-GuK) and stained as described in panel B. Note that this construct is recruited by JAM-C. Data are representative of *N* = 3 independent experiments. Scale bars: 10 μm.

To characterize the interaction between JAM-C and ZO-2 in cells in more detail, we expressed ZO-2 truncation constructs consisting of either the three PDZ domains (ZO-2/PDZ123), the SH3 and GuK domains (ZO-2/SH3-GuK), or the C-terminal region comprising the actin- and CGN-binding region (ZO-2/C-term) in CHO-JAM-C cells and analyzed their localization at JAM-C-based cell-cell contacts. Notably, none of the three constructs was recruited by JAM-C (Suppl. Fig. S1). Previous studies have shown that the interaction of JAM-A with the ZO-1 scaffold protein through PDZ domain 3 requires the SH3 domain immediately adjacent to PDZ3 ^44^. In addition, structural studies of ZO-1 have shown that PDZ3 exists in a tandem organization with the SH3 and GuK domains, forming a supramodule^45^. Since this domain organization is conserved in ZO-2^5,8^, we tested ZO-2 constructs containing the SH3 and GuK domains in addition to the PDZ domains (Fig. 4A). ZO-2 constructs containing the SH3 and GuK domains adjacent to either all three PDZ domains (ZO-2/PDZ123-SH3-GuK) or PDZ domains 2 and 3 (ZO-2/PDZ23-SH3-GuK) were efficiently recruited to JAM-C-based cell-cell junctions (Fig. 4B). In addition, the ZO-2/PDZ123-SH3-GuK construct was precipitated from lysates of transfected HEK293T cells by GST-JAM-C but not by GST-JAM-C/Δ5 (Fig. 4C). These findings suggest that the interaction of JAM-C with ZO-2 is PDZ domain-dependent but requires the SH3 and GuK domains adjacent to the PDZ domains. To test whether PDZ3 in concert with the SH3 and GuK domains is sufficient for recruitment by JAM-C, we analyzed the recruitment of the ZO-2 PSG core unit (ZO-2/PDZ3-SH3-GuK). This construct was efficiently recruited by JAM-C (Fig. 4D), strongly suggesting that JAM-C interacts with PDZ3 of ZO-2, provided that PDZ3 exists in a tandem organization with the SH3 and GuK domains. Since a construct comprising the three PDZ domains alone or a construct comprising the SH3–GuK domains alone was not recruited by JAM-C, these findings further suggest that the PSG core unit of ZO-2 forms a functional supramodule^8^.

**Fig. 5:**
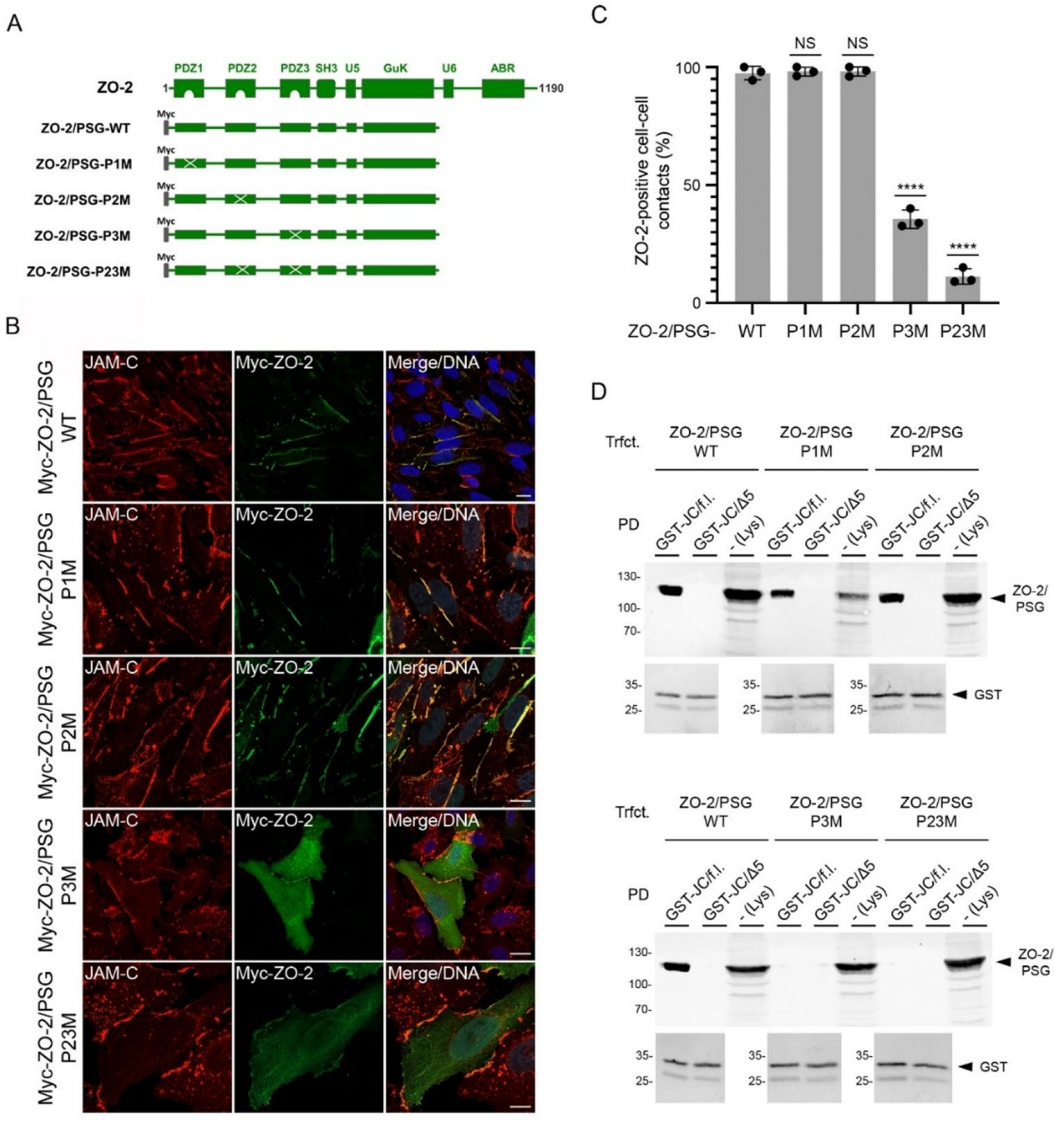
JAM-C interacts with ZO-2 through PDZ domain 3 of ZO-2. (**A**) Cartoon of ZO-2 constructs used for mapping experiments. White crosses within the PDZ domains indicate function-inactivating mutations in the GLGF motif of the respective PDZ domain. (**B**) CHO cells stably expressing mJAM-C (CHO-JAM-C) were transfected with Myc-tagged ZO-2 constructs (depicted in A) and stained with antibodies against JAM-C (red) and Myc (green). Scale bars: 10 μm. (**C**) Statistical analysis of ZO-2 constructs recruitment by JAM-C. The bar graph shows the fraction of cell-cell contacts in which Myc-ZO-2 co-localizes with JAM-C. Statistical analysis was performed with unpaired Student’s t-test. Data is derived from *N* = 3 independent experiments, each data point represents the mean value of one experiment. Data are presented as mean values ± SD. Number of analyzed cell-cell contacts: n(ZO-2/PSG-WT): 154; n(ZO-2/PSG-P1M): 156; n(ZO-2/PSG-P2M): 175; n(ZO-2/PSG-P3M): 349; n(ZO-2/PSG-P23M): 207. ****P < 0.0001. NS, not significant. (**D**) GST fusion proteins (described in the legend to Fig. 3) were incubated with lysates from HEK293T cells transfected Myc-tagged ZO-2/PSG constructs containing mutations in the GLGF motif in one or several PDZ domains (ZO-2/PSG-P1M, ZO-2/PSG-P2M, ZO-2/PSG-P3M, ZO-2/PSG-P23M). The unmutated construct (ZO-2/PSG-WT) served as positive control and is present in both gels. Bound proteins were analyzed by immunoblotting using anti-Myc antibodies. The lanes labeled “- (Lys)” contain postnuclear supernatants of transfected cells (25% of input). The amounts of GST fusion proteins used in the pulldown experiments were visualized with anti-GST antibodies (bottom panels, 10% of input). Data is representative of *N* = 3 independent experiments. Abbreviations: PD, pulldown; Trfct., transfection.

To address whether other PDZ domains contribute to the interaction with JAM-C, we analyzed the recruitment of ZO-2 constructs in which individual PDZ domains were inactivated by mutating two conserved residues within the canonical “GLGF” motif, a key feature of PDZ domains^46^ (Fig. 5A). Constructs with inactivated PDZ1 or PDZ2 (ZO-2/PSG-P1M, ZO-2/PSG-P2M, respectively) were efficiently recruited by JAM-C to cell-cell contacts (Fig. 5B, C). Inactivation of PDZ3 (ZO-2/PSG-P3M) resulted in reduced but not abolished recruitment by JAM-C (Fig. 5B, C). Inactivation of PDZ3 in combination with PDZ2 (ZO-2/PSG-P23M) almost completely abolished recruitment to JAM-C-based cell-cell contacts (Fig. 5B, C). These observations support the notion that JAM-C recruits ZO-2 predominantly through PDZ3, but also suggest a possible contribution of PDZ2. To further verify this, we performed GST pull-down experiments with lysates of HEK293T cells transfected with the ZO-2 PDZ mutant constructs. ZO-2/PSG-P1M and ZO-2/PSG-P2M were efficiently precipitated by GST-JAM-C in a PBM-dependent manner (Fig. 5D). In contrast, ZO-2/PSG-P3M as well as ZO-2/PSG-P23M were not precipitated by GST-JAM-C (Fig. 5D). Together, these observations suggest that the interaction of JAM-C with ZO-2 involves the PBM of JAM-C and the PSG core unit of ZO-2.

**Fig. 6:**
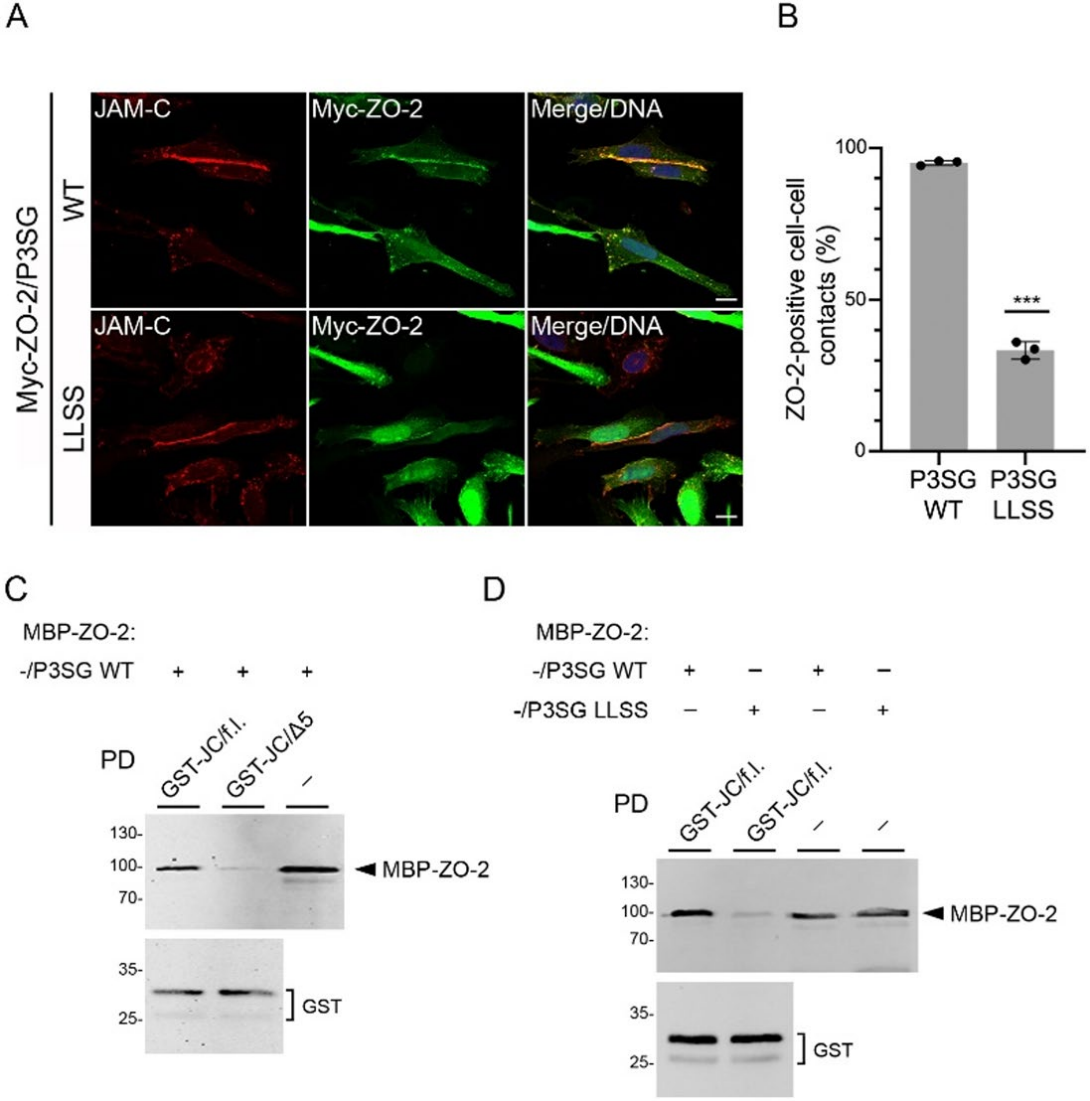
Hydrophobic residues in the SH3 domain of ZO-2 are involved in the interaction with JAM-C. (**A**) CHO cells stably expressing mJAM-C (CHO-JAM-C) were transfected with the Myc-tagged ZO-2 PSG core module, either wildtype (ZO-2/P3SG_WT) or with leucine residues Leu637 and Leu642 present in the SH3 domain mutated to serine residues (ZO-2/P3SG_LLSS). Cells were stained with antibodies against JAM-C (red) and the Myc tag (green). Scale bars: 10 μm. (**B**) Statistical analysis of ZO-2 constructs recruitment by JAM-C. The bar graph shows the fraction of cell-cell contacts in which Myc-ZO-2 co-localizes with JAM-C . Statistical analysis was performed with unpaired Student’s t-test. Data is derived from N = three independent experiments. Data are presented as mean values ± SD, each data point represents the mean value of one experiment. Number of analyzed cell-cell contacts: n(ZO-2/P3SG-WT): 208; n(ZO-2/P3SG-LLSS): 228. ***P < 0.001. (**C**,** D**) GST-pulldown experiment with recombinant MBP-ZO-2 proteins purified from E.coli bacteria. GST-JAM-C fusion proteins (GST-JC/f.l., GST-JC/∆5) were immobilized on GSH sepharose beads and incubated with recombinant, purified MBP fusion proteins consisting of the either wildtype or mutated ZO-2 PSG core unit fused to MBP (MBP-ZO-2/P3SG, MBP-ZO-2/P3SG_LLSS). Bound proteins were analyzed by immunoblotting using anti-MBP antibodies. The lanes labeled “–“ contain 50 ng of purified MBP-ZO-2/P3SG or MBP-P3SG_LLSS fusion proteins (5% of input), as indicated. Application of equal amounts of GST fusion proteins used in the pulldown experiments was controlled with anti-GST antibodies (bottom panel, 10% of input). Data is representative of *N* = 3 independent experiments. Abbreviations: MBP, maltose-binding protein; PD, pulldown.

To further corroborate this notion, we mutated amino acid residues in the SH3 domain of ZO-2 (L637 and L642). Structural studies of the ZO-1 PSG core unit complexed with a JAM-A peptide have shown that the homologous residues (L549 and L554 in ZO-1) interact with a residue in the JAM-A PBM (F298)^44^. Mutating L637 and L642 to serine in the ZO-2 PSG core unit impaired recruitment to cell-cell contacts in CHO-JAM-C cells (Fig. 6A, B).

These findings support a contribution of the SH3 domain and suggest that the interaction of ZO-2 with JAM-C is direct. To test this, we performed in vitro binding assays using recombinant proteins. GST-JAM-C fusion proteins were immobilized on glutathione Sepharose beads and incubated with a recombinant MBP–ZO-2 fusion protein consisting of the PSG core unit (MBP–ZO-2/P3SG). The ZO-2 fusion protein was detected in GST-JAM-C/f.l. but not in GST-JAM-C/Δ5 precipitates (Fig. 6C), confirming that the interaction is direct. Mutating L637 and L642 to serine in MBP–ZO-2/P3SG (MBP–ZO-2/P3SG_LLSS) strongly reduced binding to GST-JAM-C (Fig. 6D), indicating that these residues in the SH3 domain contribute to the direct interaction with JAM-C.

Together, these observations demonstrate that JAM-C directly interacts with ZO-2 via the PBM of JAM-C and both the PDZ3 and SH3 domains of ZO-2, which are part of the ZO-2 PSG core unit. They further support the notion that the PSG core unit of ZO-2 forms a functional supramodule, as previously shown for ZO-1^44,45^.

## JAM-C recruits cingulin to cell-cell junctions

**Fig. 7:**
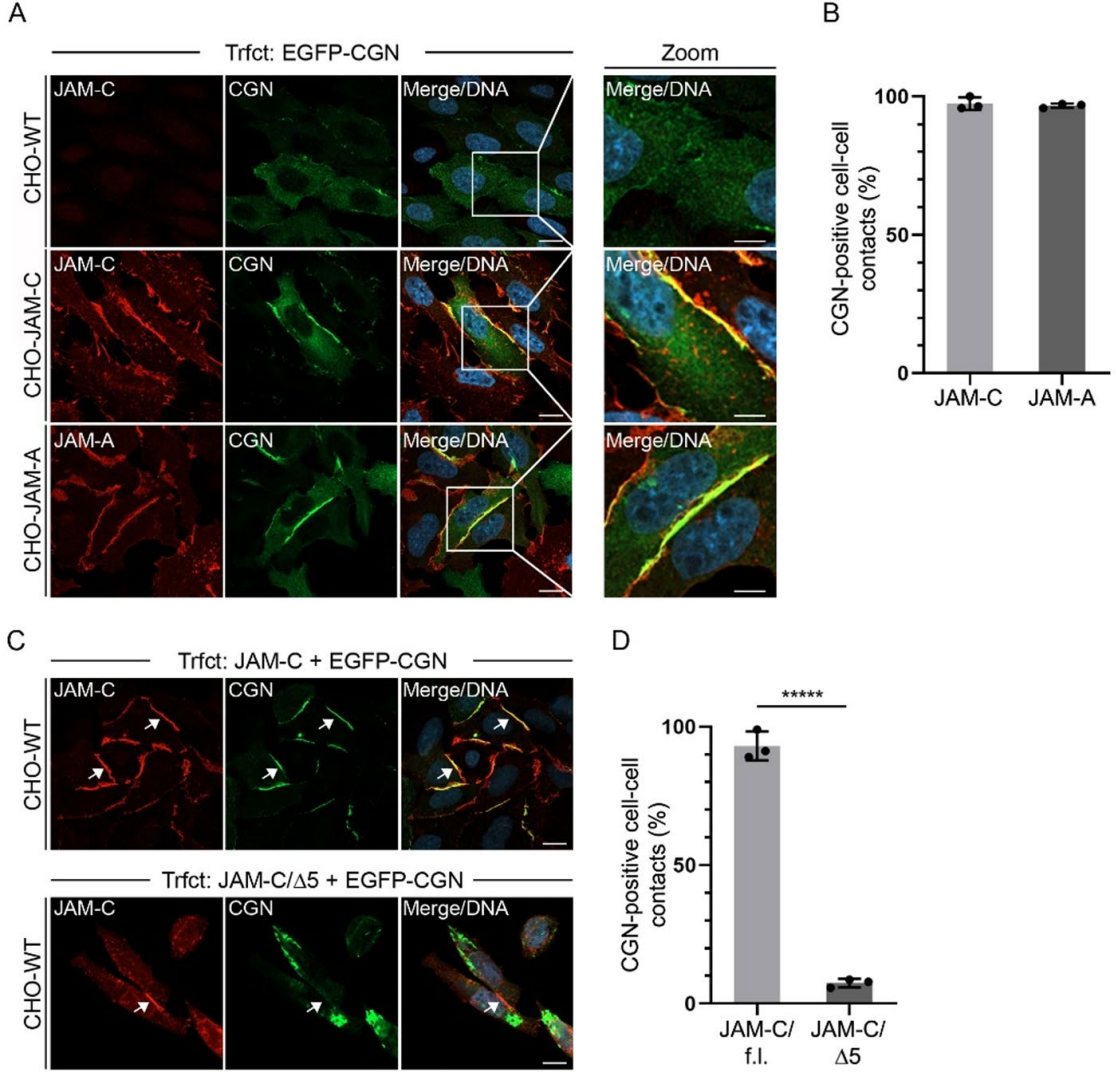
JAM-C recruits Cingulin to cell-cell contacts through its PDZ domain binding motif. (**A**) Wildtype CHO cells (CHO-WT) or CHO cells stably expressing either mJAM-C (CHO-JAM-C) or mJAM-A (CHO-JAM-A) were transfected with EGFP-tagged Cingulin (EGFP-CGN) and stained with antibodies against JAM-C or JAM-A as indicated. The right panels (Zoom) show magnifications of the insets marked by white squares in the Merge/DNA images. Scale bars: 10 μm (regular images), 5 μm (zoomed insets). (**B**) Statistical evaluation of CGN recruitment by JAM-A and JAM-C. The bar graph shows the fraction of EGFP-CGN-positive cell-cell contacts in JAM-C- and JAM-A-expressing CHO cells. Data are derived from *N* = 3 independent experiments, each data point represents the mean value of one experiment. Data are presented as mean values ± SD. Number of analyzed cell-cell contacts: n(JAM-C–CGN): 202; n(JAM-A–CGN): 210; (**C**) CHO WT cells were transiently co-transfected with EGFP-CGN and either JAM-C full length (JAM-C/f.l.) or a JAM-C mutant lacking the PDZ domain binding motiv (JAM-C/Δ5). Cells were stained with antibodies against JAM-C. Scale bars: 10 μm. (**D**) Statistical analysis of CGN recruitment by JAM-C/f.l. and JAM-C/Δ5. The bar graph shows the fraction of cell-cell contacts in which EGFP-CGN co-localizes with either JAM-C/f.l. or JAM-C/Δ5. Statistical analysis was performed with unpaired Student’s t-test. Data are derived from *N* = 3 independent experiments, each data point represents the mean value of one experiment. Data are presented as mean values ± SD. Number of analyzed cell-cell contacts: n(JAM-C/f.l.): 279; n(JAM-C/Δ5): 131. *****P < 0.00001. Abbreviations: CGN, Cingulin; Trfct, transfection.

ZO-2 acts as a scaffold protein at the TJs which interacts with numerous proteins including integral membrane proteins such as claudins and occludin, and other scaffolding and adapter proteins such as ZO-1, cingulin (CGN) and CGN-like 1 (CGNL1)^5,47^. CGN is an adapter protein that binds various regulators of Rho family GTPases and recruits these to TJs through its interaction with ZO proteins^5^. Given that CGN interacts with ZO-2 through a region in the C-terminus of ZO-2 that does not overlap with the PSG core module^5^, we hypothesized that JAM-C recruits CGN to cell-cell junctions in CHO cells. CGN was efficiently recruited to cell-cell junctions in CHO cells expressing either JAM-C or JAM-A whereas no recruitment was observed in CHO-WT cells (Fig. 7A, B). Deletion of the PBM in JAM-C abolished the recruitment of CGN (Fig. 7C, D) suggesting that its recruitment by JAM-C is indirect and mediated by a PDZ domain protein. These observations indicate that CGN exists in a complex with JAM-C most likely through an indirect association via a JAM-C-interacting scaffolding protein such as ZO-1 or ZO-2.

## JAM-C expression is suppressed by JAM-A in MDCKII cells

**Fig. 8:**
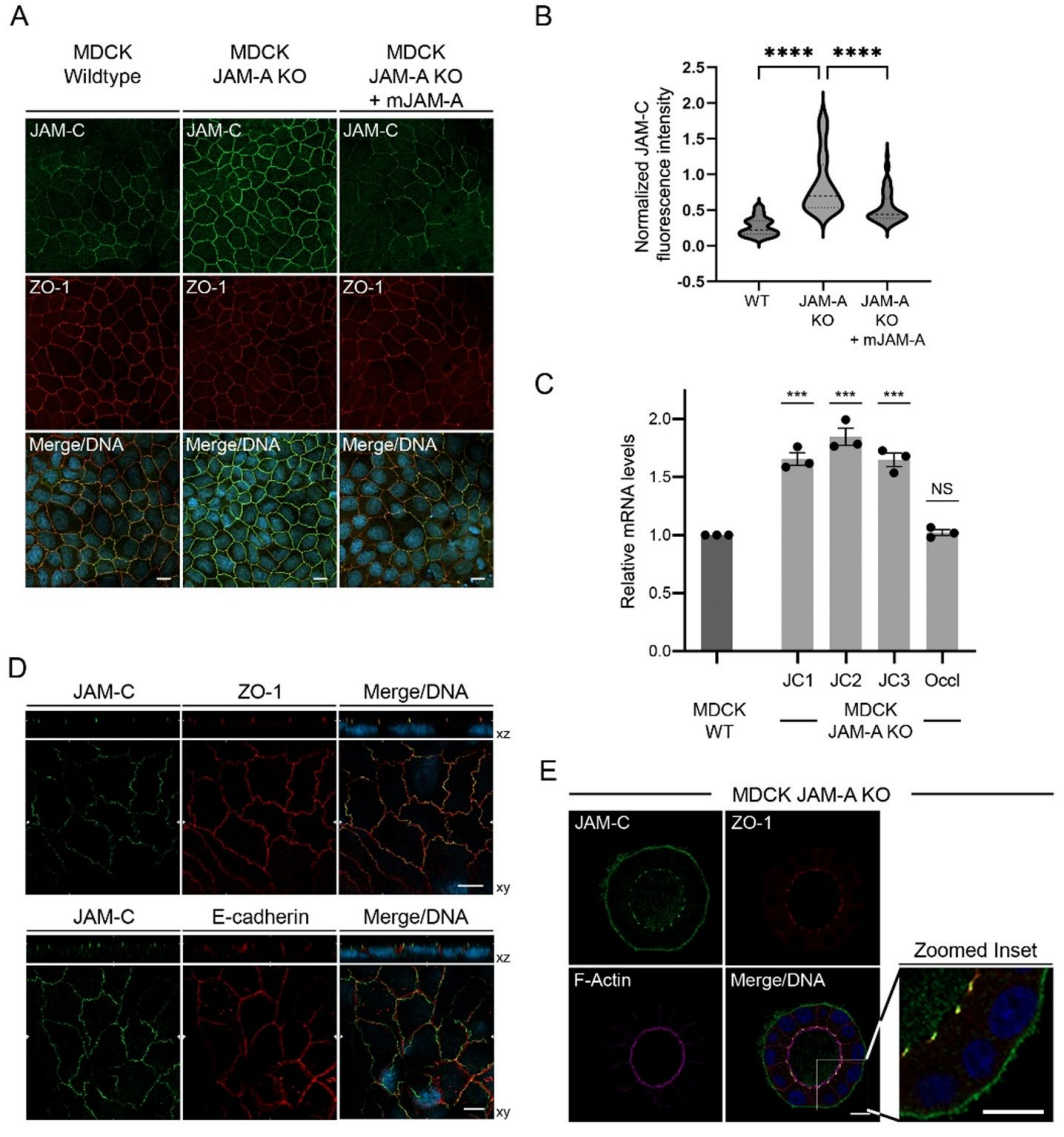
JAM-C is upregulated and localizes to TJs in JAM-A knockout MDCKII cells. (**A**) Wild-type MDCKII cells, JAM-A KO MDCKII cells or JAM-A KO MDCKII cells ectopically expressing mJAM-A were grown to confluency and stained with antibodies against JAM-C and ZO-1. Scale bars: 10 μm. (**B**) Statistical analysis of JAM-C localization at cell-cell contacts in confluent cells. The JAM-C and ZO-1 fluorescence intensities were analyzed using Imaris software. The data show ratios of JAM-C fluorescence at cell-cell contacts relative to ZO-1 fluorescence intensity and is depicted as normalized JAM-C fluorescence intensity. Statistical analysis was performed with Kruskal-Wallis test and Dunn’s post hoc multiple comparisons test. Data were obtained and data points were pooled from at least 11 randomly chosen fields of view (FOV) per experiment derived from *N* = 3 independent experiments (WT cells: 59 FOV; JAM-A KO cells: 57 FOV; JAM-A KO cells expressing mJAM-A: 55 FOV). Data are presented as Violin plot. Broken lines indicate mean values, dotted lines indicate first and third quartiles. ****p < 0.0001. (**C**) qRT-PCR analysis of mRNA levels of JAM-C (JC) and Occludin (Occl) in JAM-A KO MDCKII cells. mRNA levels were normalized to GAPDH. Data show normalized mRNA levels in JAM-A KO MDCKII cells relative to WT MDCKII cells (MDCKII WT). JAM-C mRNA was analyzed with three primer sets (JC1, JC2, JC3). Statistical analysis was performed with unpaired Student’s t-test. Data are presented as mean values ± SEM from triplicate samples derived from *N* = 3 independent experiments.***P < 0.001. NS, not significant. (**D**) JAM-A KO MDCKII cells were grown on polycarbonate filters to induce polarization and stained with antibodies against JAM-C and ZO-1 (top panels) or against JAM-C and E-cadherin (bottom panels). Cells were analyzed by confocal microscopy. Rectangles depict XZ projections of the XY projections at positions indicated by small arrowheads. Scale bars: 5 μm. (**E**) JAM-A KO MDCKII cells were grown in 3D matrigel matrices for 4–6 days and stained as indicated. Scale bars: 10 μm.

Several examples of cross-talk between adhesion receptors localized at cell-cell contacts exist. In endothelial cells, VE-cadherin regulates the expression of the adherens junction (AJ)-localized N-cadherin and, vice versa, N-cadherin regulates VE-cadherin expression^48,49^. A similar mutual regulation has been described for the integrin family of cell-matrix adhesion receptors^50^. To test a possible regulation of JAM-C by JAM-A in epithelial cells we used MDCKII cells with a CRISPR/Cas9-mediated inactivation of the JAM-A gene (MDCKII/JAM-A KO)^51,52^ (Suppl. Fig. S2) and analyzed the localization of endogenous JAM-C at cell-cell contacts using a polyclonal antibody generated against canine JAM-C (see Methods for details). In MDCKII wildtype cells, JAM-C was localized weakly at cell-cell contacts, with a prominent variability in fluorescence intensity between individual cells (Fig. 8A). In MDCKII/JAM-A KO cells the JAM-C fluorescence intensity at cell-cell contacts was strongly increased and more homogeneously distributed among cells. Ectopic expression of mJAM-A suppressed junctional localization of JAM-C in MDCKII/JAM-A KO cells (Fig. 8A, B). To test if the upregulation of JAM-C was dependent on the state of confluency, MDCKII cells were seeded at low density and fixed at subconfluency. As observed in confluent cells, JAM-C was significantly upregulated in MDCKII/JAM-A KO cells, and upregulation of JAM-C was suppressed by ectopic expression of mJAM-A (Suppl. Fig. S3). To gain insight into potential mechanisms underlying JAM-C upregulation, we analyzed JAM-C mRNA levels by qRT-PCR. JAM-C mRNA levels were significantly upregulated in JAM-A KO MDCKII cells (Fig. 8C), suggesting that JAM-A suppresses JAM-C expression at the level of mRNA. To test if JAM-C localized at TJs, MDCKII/JAM-A KO cells were cultured on transwell filters to promote polarization. JAM-C immunofluorescence overlapped with ZO-1 at the apical region of cell-cell contacts but was absent at E-cadherin-positive regions at the lateral membrane domain indicating a TJ-specific localization of JAM-C (Fig. 8D). JAM-C also colocalized with ZO-1 at the apical cell-cell contact region when cells were grown to three-dimensional cysts (Fig. 8E). Together, these findings indicated that JAM-A suppresses JAM-C mRNA expression in MDCKII cells and that endogenous JAM-C is exclusively localized at TJs in these cells. The increased expression of JAM-C suggested the possibility of an enhanced ZO-2 recruitment to cell-cell contacts. However, ZO-2 localization at cell-cell contacts was reduced in JAM-A KO cells (Suppl. Fig. S4), suggesting that the localization of ZO-2 at cell-cell contacts is influenced by additional factors, possibly by other interaction partners such as claudins, ZO-1, or the actin cytoskeleton, whose localization might be altered in JAM-A KO cells^5^. Together, these data indicate that JAM-A suppresses JAM-C expression in polarized epithelial cells and that endogenous JAM-C localizes to TJs.

## Discussion

In this study, we identify the TJ-associated transmembrane protein JAM-C as a direct binding partner of the scaffolding protein ZO-2. The interaction is mediated by the C-terminal PDZ-binding motif (PBM) of JAM-C and involves both the PDZ3 and SH3 domains of ZO-2. Two hydrophobic residues within the ZO-2 SH3 domain are critical for complex formation, defining a previously unrecognized structural determinant of this interaction. JAM-C efficiently recruits ZO-2 to cell-cell contacts. JAM-C also promotes the accumulation of CGN at cell–cell junctions, most likely indirectly via ZO-2 or its homologue ZO-1. Moreover, JAM-C expression is negatively regulated by JAM-A in polarized epithelial cells, in which endogenous ZO-2 is strictly confined to TJs. Together, these findings position JAM-C as a regulatory component of the ZO protein scaffold at TJs.

A key finding of this study is that the PDZ domains of ZO-2 alone are insufficient to mediate JAM-C binding. Although recruitment of ZO-2 depends on the C-terminal PDZ-binding motif (PBM) of JAM-C, a construct comprising all three PDZ domains fails to localize to cell-cell contacts in response to JAM-C, indicating that additional structural elements are required. Insights from the interaction of JAM-A with ZO-1, which involves PDZ3 and residues within the adjacent SH3 domain^41,44^, prompted us to examine a similar contribution of the SH3 domain in ZO-2. Structural analysis of the ZO-1 PSG module in complex with the C-terminal peptide of JAM-A demonstrated that a conserved phenylalanine within the PBM engages two hydrophobic residues in the SH3 domain (L_549_YNGKL_554_)^44,53^. Notably, the corresponding residues are conserved in JAM-C and ZO-2 (L_637_YDGKL_642_). Consistent with this model, substitution of two hydrophobic residues within the ZO-2 SH3 domain with polar residues markedly impaired JAM-C–dependent recruitment in cells and strongly reduced complex formation in vitro, demonstrating that efficient binding requires cooperative engagement of PDZ3 and the adjacent SH3–GUK module. These findings support the concept that the PSG region of ZO-2 functions as an integrated supramodule. Together with the JAM-A–ZO-1 interaction^44,45^ and the Crumbs–Pals1 interaction^54^, our data establish the JAM-C–ZO-2 interaction as a further example of an integral^55^ membrane protein engaging a MAGUK PSG supramodule.

Our recruitment assays in CHO cells indicate that, among the three PDZ domains of ZO-2, PDZ3 is not solely responsible for the interaction with JAM-C and that PDZ2 contributes to this association. This conclusion is based on experiments with PDZ domain mutants. Although inactivation of PDZ3 markedly reduced recruitment to JAM-C-based cell-cell contacts, we observed significant junctional localization of this mutant. Only simultaneous inactivation of PDZ2 and PDZ3 completely abolished junctional recruitment. One interpretation is that JAM-C engages not only PDZ3 within the PSG supramodule but also PDZ2. Consistent with this notion, multi-PDZ proteins can use more than one PDZ domain to bind the same ligand. For example, the JAM-related IgSF member TMIGD1 associates with PDZ1 and PDZ3 of the polarity protein Scribble^56^. Alternatively, the residual recruitment observed with the PDZ3 mutant may reflect indirect association via endogenous ZO-1 and/or ZO-2. The PDZ2 domains of both ZO-1 and ZO-2 are capable of homo- and heterodimerization through domain swapping^57,58^. This mechanism involves reciprocal β-strand exchange between monomers, generating dimers with two functional PDZ domains^57,59^. Since the PDZ3 mutant construct used here retains an intact PDZ2 domain, it likely interacts with endogenous ZO proteins via PDZ2-mediated domain swapping, resulting in indirect recruitment to JAM-C-containing junctions. Importantly, several lines of evidence argue that PDZ3 within the PSG supramodule is the principal determinant of direct JAM-C binding: (i) the PSG core unit is sufficient for junctional localization; (ii) a ZO-2 construct with inactivated PDZ3 is not precipitated by GST-JAM-C; and (iii) the recombinant PSG core unit directly binds GST-JAM-C in vitro. Together, these findings support a model in which PDZ3 of ZO-2 mediates the direct interaction with JAM-C.

While JAM-C recruited EGFP-ZO-1 and EGFP-ZO-2 with similar efficiencies in CHO cells, it showed a strong preference for EGFP-ZO-2 over EGFP-ZO-1 in GST-pulldown assays from transfected HEK293T cells. This finding could possibly be explained by different compositions of the ZO protein complexes in the two cell types. PDZ domain interactions of scaffolding proteins with ligands are often regulated by cooperative ligand binding. As one example, the MAGUK protein PSD-95 undergoes a conformational change in response to PDZ domain ligand binding, resulting in coupling of PDZ3 to the SH3 and GuK domains and binding of microtubule-associated protein 1 A to the GuK domain^60,61^. The presence of PDZ ligands in CHO cells could selectively promote PSG module formation and JAM-C binding. Since both CHO cells and HEK293T cells express ZO-1 and ZO-2 endogenously (Fig. 1, Suppl. Fig. S5) differences in endogenous ZO-1 or ZO-2 expression cannot account for observations.

Our observations could possibly also be explained by the nature of the bond that mediates the interaction between JAM-C and ZO-1. A recent study shows that the interaction of JAM-A with the PSG module of ZO-1 becomes weaker in the presence of mechanical load^62^. A similar type of bond between JAM-C and ZO-1 would explain our observation that a JAM-C – ZO-1 interaction is not demonstrated in pulldown assays where protein complexes immobilized on glutathione sepharose beads are exposed to hydrodynamic shear forces during washing and centrifugation steps. Such an interaction would explain observations of very weak or even absent binding of ZO-1 to JAM-A and JAM-C in pulldown and immunoprecipitation assays^36,43^. It will be important to directly compare the binding affinities of the JAM-C intracellular domain for the PSG modules of ZO-1 and ZO-2 in the absence and presence of mechanical load.

One limitation of our study is that it cannot provide direct evidence that JAM-C recruits ZO-2 to TJs in MDCKII cells. In contrast to CHO cells, MDCKII cells express multiple potential ZO-2 binding partners at TJs, including JAM-A, occludin, claudins, and ZO-1^35,43,55,63,64^, suggesting that depletion of a single binding partner does not affect ZO-2 at TJs. In support of this, simultaneous depletion of claudin-1, -2, -3, -4, and − 7 and JAM-A resulted in only rare ZO-2 discontinuities at cell-cell junctions^51^. RNAseq experiments revealed that JAM-C mRNA expression levels are much lower than any of the other TJ membrane proteins tested, including JAM-A, CAR, occludin, claudin-1, and claudin-2 (Table S1), making it unlikely that JAM-C is responsible for the localization of ZO-2 at TJs. Most likely, ZO-2 is incorporated into stable protein complexes through its interactions with multiple integral membrane proteins and scaffolding proteins, at least in mature cell-cell contacts. However, it is conceivable that JAM-C contributes to the junctional localization of ZO-2 during junction formation. In human retinal pigment epithelial cells, JAM-C is localized at primordial, punctate junctions, and its depletion delays the recruitment of ZO-1 and N-cadherin to cell-cell contacts^35^, which suggests a role in junction formation and maturation. It is therefore possible that JAM-C regulates the recruitment of ZO-2 at early stages of cell-cell contact formation, when other potential binding partners such as occludin and claudins are not present yet^65^.

Our study reveals similarities but also differences between JAM-A and JAM-C in ZO protein binding. Both proteins directly interact with ZO-1^36,41,42,44,62^ and ZO-2^43,55^ through their PBM. As an important difference, however, the interaction of JAM-A with ZO-2 involves PDZ2^43,55^, whereas the interaction of JAM-C with ZO-2 involves PDZ3 and the adjacent SH3 domain as part of the PSG core module (this study). This raises the possibility that ZO-2 can simultaneously interact with JAM-A and JAM-C, thereby possibly influencing the conformation of ZO-2 and its ability to engage in higher-order complexes.

Our findings have implications for important aspects of TJ biology, including mechanosensation, mechanical resilience, and LLPS. TJs are under tensile stress which is sensed and translated into cellular responses by ZO proteins through conformational adaptations^14–16^. In addition, tensile stress leads to transient local breaches that are rapidly sealed by actin polymerization and actomyosin contractility^66,67^. The absence of integral membrane proteins including claudins, JAM-A, and CAR exacerbates breach formation^67^, indicating that these adhesion molecules contribute to the physical strength of cellular interaction. Finally, TJ formation through LLPS of ZO proteins is strongly enhanced by claudins and JAM-A^23^. By its interaction with ZO proteins, JAM-C could contribute to all of these aspects. Future studies will be required to delineate more precisely the specific role of JAM-C during these processes.

## Materials and methods

### Cell culture and transfections

HEK293T cells (ATCC-CRL-2316) were grown in DMEM containing 10% FCS, 1% NEAA, 2 mM L-Glu, 100 U/ml Pen/Strep. CHO^dhfr−^ cells (ATCC-CRL-2316)^68^ were obtained from Dietmar Vestweber (MPI Münster, Münster, Germany) and cultured in αMEM medium (Pan Biotch #P04-21500) containing 10% FCS, 2 mM glutamine, 100 U/ml Pen/Strep. CHO^dhfr−^ cells stably transfected with either mJAM-A or mJAM-C have been described previously^36,37^. Briefly, cells were grown in αMEM medium supplemented with either blasticidin (7 µg/ml, JAM-A CHO) or G418 (500 µg/ml, JAM-C CHO). Note that JAM-C CHO cells were generated using a S281A mutant of JAM-C because phosphorylation of JAM-C at Ser281 prevents cell-cell contact localization^33,36^. MDCKII cells^69^ were obtained from the European Collection of Cell Cultures (ECACC #00062107) or were provided by Masayuki Murata (University of Tokyo, Tokyo, Japan). MDCKII cells with CRISPR/Cas9-mediated inactivation of the JAM-A gene (JAM-A KO MDCKII)^51^ were cultivated in DMEM high glucose medium (Sigma-Aldrich (SA) #D5671) containing 10% FCS, 2 mM glutamine, 100 U/ml Pen/Strep. JAM-A KO MDCKII with tetracycline-regulated expression of mouse JAM-A (mJAM-A)^52^ were cultured in DMEM medium additionally containing 1 µg/ml puromycin. Expression of mJAM-A was induced by addition of 2 µg/ml doxycycline. To analyze JAM-C localization in polarized epithelial cells, MDCKII cells were grown on polycarbonate filters (0.45 μm pore size, Corning #3413) coated with growth factor reduced basement membrane Corning Matrigel matrix (Merck #CLS354230) in 24-well tissue culture dishes. Transient transfections of cDNAs were performed using X-Fect (Xfect™ Transfection Reagent, TaKaRa Bio Europe SAS, #631318, Saint-Germain-en-Laye, France), according to the manufacturer’s instructions. All cell lines were routinely tested and found to be negative for mycoplasma contamination.

### Expression vectors

Human ZO-2 constructs in pKE081myc (N-terminal myc tag,^41^): ZO-2/PDZ1-3 (PDZ domains 1,2,3; AA1-606); ZO-2/SH3-GuK (SH3 and GuK domains, AA591-889); ZO-2/Cterm (C-terminus, AA877-1190); ZO-2/P123SG_WT (PDZ1,2,3, SH3, GuK domains, AA1-892); ZO-2/P23SG (PDZ2,3, SH3, GuK domains AA281-892); ZO-2/P3SG (PDZ3, SH3, GuK domains, AA489-892); ZO-2/P123SG_P1M (AA1-892, F_44_A_G_45_E); ZO-2/P123SG_P2M (AA1-892, Y_319_A_G_320_E); ZO-2/P123SG_P3M (AA1-892, V_521_A_G_522_E); ZO-2/P123SG_P23M (AA1-892, Y_319_A_G_320_E _V_521_A_G_522_E); ZO-2/P3SG_LLSS (AA489-892, L_637_S_L_642_S). Human ZO-2 in pMAL-c2X: ZO-2/P3SG (AA489-892); ZO-2/P3SG_LLSS (AA489-892, L_637_S_L_642_S). Human ZO-2 in pEGFP-C3 (Addgene plasmid #27422; http://n2t.net/addgene:27422; gift from Marius Sudol^70^). Murine ZO-1 in pEGFP-C1 (kindly provided by Dr. Karl Matter (University College London, UK) and Dr. Junichi Ikenouchi (Kyushu University, Japan). Canine Cingulin in pcDNA3: EGFP-Cingulin (AA1196)-Myc. Murine JAM-C in pcDNA3: mJAM-C/f.l. (AA1-310, S_281_A), mJAM-C/∆5 (AA1-305, S_281_A). Murine JAM-C in pGEX plasmid vectors: mJAM-C-CP (cytoplasmic part, AA264-310) in pGEX-5X-2; mJAM-C-CP∆5 (AA264-305) in pGEX-6P-2.

### Antibodies and reagents

The following antibodies were used in this study: mouse mAb anti-Myc 9E10^71^ (IF 1:500, WB 1:500); goat pAb anti c-Myc (Santa Cruz #sc-789-G; WB 1:500); mouse mAb anti-GFP (Takara #632375, WB 1:1000); alpaca anti-GFP nanobody (Nano-Tag #N0310, used for IP); rabbit pAb anti-MBP (New England Biolabs (NEB) E8030S; WB 1:5000); mouse mAb anti-GST (ThermoFisher #13-6700; WB 1:500); mouse mAb anti-ZO-1 (Invitrogen #33-9100; 1:500 in regular ICC (IF), 1:250 in MDCK cyst assays (IF); rat mAb anti-ZO-1 R40.64 (IF 1:250); rabbbit pAb anti-ZO-2 (Invitrogen #71-1400; IF 1:250); rabbit pAb anti-Par-3 (Millipore #07-330; IF 1:250); mouse mAb anti-E-cadherin (BD Biosciences #610181; 1:100 in regular ICC (IF), 1:50 in MDCK cyst assays (IF); rabbit pAb anti-N-cadherin (proteintech #22018-1-AP; IF 1:200); mouse mAb anti-β-catenin (BectonDickinson #610154, IF 1:250); rat mAb anti-mJAM-A^72^; IF dil 1:300); rabbit anti-cJAM-A pAb Affi831 (IF 1:500) has been described before^73^; goat anti-mJAM-C (R&D Systems #AF1213; WB 1:500, IF 1:333). Rabbit anti-mJAM-A pAb Affi1165, rabbit anti-mJAM-C Affi1166, rabbit anti-mJAM-C Affi1840, and rabbit anti-canine JAM-C pAb Affi1917 were generated by immunizing rabbits with fusion proteins consisting of the extracellular domain of the respective JAM protein (mouse JAM-A: Gene ID 23929; mouse JAM-C: Gene ID 83964; canine JAM-C: Gene ID 489271) fused to the Fc region of human IgG, as described previously^74^. The antibodies were affinity-purified by adsorption at the antigen covalently coupled to cyanogen bromide (CNBr)-activated sepharose beads (Amersham Biosciences Europe, Freiburg, Germany). Antibodies directed against the Fc part were depleted by adsorption at human IgG coupled to CNBr-activated sepharose beads. Affinity-purified antibodies were dialyzed against PBS. Affi1165 was used at 1:500 (IF), Affi1166 was used at 1:500 (IF), Affi1917 was used at 1:200 in regular ICC (IF), 1:100 in MDCK cyst assays (IF). Secondary antibodies and fluorophore-conjugated antibodies: Fluorophore-conjugated antibodies for Western blotting: IRDye 800CW donkey anti-rabbit IgG (LI-COR Biosciences #926-32213, WB 1:10.000), IRDye 680CW donkey anti-mouse IgG (LI-COR Biosciences #926-68072, WB 1:10.000). Fluorophore-conjugated secondary antibodies for ICC: donkey anti-mouse IgG (H + L) Alexa Fluor 594 (ThermoFisher Scientific #A-21203); donkey anti-rabbit IgG (H + L) Alexa Fluor 594 (ThermoFisher Scientific #A-21207); donkey anti-rabbit IgG(H + L) Alexa Fluor 488 (ThermoFisher Scientific #A-21206); donkey anti-mouse IgG (H + L) Alexa Fluor 488 (Dianova/Jackson ImmunoResearch Europe Ltd #715-545-150); donkey anti-mouse IgG (H + L) Alexa Fluor 647 (Dianova/Jackson ImmunoResearch Europe Ltd #715-605-150). All Alexa Fluor-conjugated secondary antibodies used in IF experiments were used at a dilution of 1:800. Alexa Fluor 647-Phalloidin (Thermo Fisher Scientific, Invitrogen #A22287; dil 1:200).

### Immunocytochemistry and immunofluorescence microscopy

For immunofluorescence microscopy, cells were grown on Cultrex Basement Membrane Extract (Cultrex BME, R&D Systems #3432-010-01)-coated glass slides. Cells were washed with PBS and fixed with either 4% paraformaldehyde (PFA, Sigma-Aldrich) for 10 min at RT. To detect intracellular proteins, PFA-fixed cells were incubated with PBS containing 0.5% Triton X-100 for 10 min. Cells were washed with 100 mM glycine in PBS (3 × 10 min, RT), blocked for 1 h in blocking buffer (PBS, 10% FCS, 0.2% Triton X-100, 0.05% Tween-20, 0.02% BSA) and then incubated with primary antibodies in blocking buffer for 1 h at room temperature (RT) or overnight at 4 °C. After incubation, cells were washed with PBS (3 × 5 min, RT) and incubated with fluorochrome (AlexaFluor488, AlexaFluor568, AlexaFluor594, AlexaFluor647)-conjugated, highly cross-adsorbed secondary antibodies (Invitrogen) for 2 h at RT protected from light. DNA was stained with 4,6-diamidino-2-phenylindole (DAPI, SA). Samples were washed three times with PBS and mounted in fluorescence mounting medium (Mowiol 4–88, SA #81381, or Fluoromount G, ThermoFisher Scientific #00-4958-02). Immunofluorescence microscopy was performed using the confocal microscope LSM 800 Airyscan (Carl Zeiss, Jena, Germany) equipped with the objective Plan-Apochromat x 63/1.4 oil differential interference contrast (Carl Zeiss). Image processing and quantification was performed using ZEN 2012 (Carl Zeiss), ImageJ (National Institutes of Health, Bethesda, MD), and Imaris (Bitplane, Version 9.1.2) softwares.

### Cell junction analysis

Analysis of construct recruitment by JAM-C or JAM-C/∆5 was performed by visual identification of cell-cell contact sites. EGFP-construct- or Myc-construct-transfected cells were identified based on the cytoplasmic EGFP or Myc fluorescence signal. JAM-C-positive cell-cell contacts were counted positive for the transfected constructs when the EGFP- or Myc-based fluorescence signals co-localized with the JAM-C-based fluorescence along the cell-cell contact sites. For quantification of cell-cell contact localization of endogenous proteins and transfected constructs, at least 50 cell-cell contacts per condition and per experiment were analyzed. Data is represented as fraction of cell-cell contacts positive for the transfected construct. Data is derived from at least 3 independent experiments. Statistical analysis was performed using unpaired Student`s t test, data is plotted as means ± standard deviation (SD). P-values: *P < 0.05, ****P < 0.001. Analysis of JAM-C localization at cell-cell contacts in JAM-A KO MDCKII cells was performed using Imaris software. Based on the ZO-1 fluorescence signal a surface mask for cell-cell contacts was generated. Background structures that were incorrectly identified during automated segmentation were manually removed. The mean fluorescence intensities of ZO-1 and JAM-C signals were measured in each surface. Data is presented as JAM-C : ZO-1 fluorescence intensity ratio. At least 11 fields of view per condition and per experiment were analyzed. Data is derived from *N* = 3 independent experiments. Data was analyzed using GraphPad Prism. To test for statistical significance, a Kruskal-Wallis-test and a Dunn’s multiple comparisons test were performed.

### Immunoprecipitation and Western blot analysis

For immunoprecipitations (IPs), cells were lysed in lysis buffer (50 mM Tris-HCl (pH 7.5), 150 mM NaCl, 1% Nonidet P-40 (AppliChem, Darmstadt, Germany), 1% Halt Protease Inhibitor cocktail (Thermo Scientific #78429) for 1 h with overhead rotation at 4 °C followed by centrifugation (15.000 rpm, 20 min at 4 °C). Postnuclear supernatants were precleared by incubation with protein A– or protein G–Sepharose beads (GE Healthcare, Solingen, Germany), followed by incubation with 2 µg of antibodies coupled to protein A– or protein G–Sepharose beads overnight at 4 °C. Beads were washed four times with lysis buffer, bound proteins were eluted by boiling in 1x SDS-sample buffer/150 mM DTT. Eluted proteins were separated by SDS–PAGE and analyzed by Western blotting with near-infrared fluorescence detection (Odyssey Infrared Imaging System Application Software Version 3.0 and IRDye 800CW-conjugated antibodies; LI-COR Biosciences, Bad Homburg, Germany). All IP and Western blot experiments shown in this study are representative for at least three independent experiments.

### Generation of recombinant GST- and MBP-fusion proteins

The generation of recombinant of Glutathion-S-Transferase (GST) and Maltose-binding protein (MBP) fusion proteins was performed essentially as described previously^41^. Briefly, the transformed bacteria (E.coli BL21, GE-Healthcare) were grown at 30 °C in LB medium/0.2% glucose to A_600_ = 0.5. Expression of recombinant proteins was induced with 0.25 mM isopropyl-β-D- thiogalactopyranoside (IPTG, Calbiochem #420322) for 2 h. The bacteria were harvested by centrifugation, resuspended in 25 ml lysis buffer (PBS for GST fusion proteins; column buffer, i.e. 20 mM Tris-HCl (pH7.4), 0.2 M NaCl, 1 mM EDTA, for MBP fusion proteins) supplemented with Halt protease inhibitors (ThermoScientific #78429), and lysed by passaging through a French pressure cell. The cell lysates were centrifuged (Sorvall JA 25.50 rotor, 30 min, 10.000 rpm) and filtrated through a 0.45 μm filter. GST and MPB fusion proteins were isolated from the raw lysates by affinity chromatography on glutathione (GSH) sepharose resin (glutathione-Sepharose 4B beads, Cytiva #17-0756-0) and amylose resin (New England Biolabs (NEB) #E8021L), respectively. The protein solutions were dialyzed against PBS, adjusted to 50% (w/v) glycerol and stored at -20 °C. Purified proteins were analyzed by SDS-PAGE and Coomassie Brilliant Blue staining.

### GST pulldown experiments

In vitro binding experiments were performed with recombinant GST-JAM-C fusion proteins purified from *E.coli* and immobilized on GSH-Sepharose 4B beads. For the analysis of direct protein interactions in vitro, recombinant MBP-ZO-2 constructs were incubated with 3 µg of immobilized GST fusion proteins for 1 h to overnight at 4 °C under constant agitation in buffer B (10 mM Hepes-NaOH (pH7.4), 100 mM KCl, 1 mM MgCl_2_, 0.1% Triton X-100). After 4 washing steps in buffer B bound proteins were eluted by boiling for 5 min in SDS sample buffer, subjected to SDS-PAGE and analyzed by by Western blotting with near-infrared fluorescence detection. To study interactions of recombinant proteins with proteins expressed in cells, cellular lysates were incubated with 3 µg of immobilized GST fusion proteins and incubated overnight at 4 °C under constant agitation. After 4 washing steps in buffer B bound proteins were eluted by boiling for 5 min in SDS sample buffer, subjected to SDS-PAGE and analyzed by Western blotting with near-infrared fluorescence detection. All in vitro binding experiments shown in this study are representative of at least three independent experiments.

### RNA isolation and reverse transcription

Total RNA was isolated from MDCKII cells using the QIAshredder (Qiagen #79656) and RNeasy Mini Kit (Qiagen #74106) according to the manufacturer’s instructions (QIAGEN, Hilden, Germany). 1st strand cDNA synthesis was performed using the High Capacity cDNA Reverse Transcription Kit (ThermoFisher #4368814) according to the manufacturer’s instructions. 1 µg of total RNA was reverse transcribed in a volume of 20 µl.

### Quantitative real-time PCR

Quantitative Real-Time PCR (qRT-PCR) was performed using iTaq™ Universal SYBR^®^ Green Supermix (Bio-Rad, Hercules, CA, USA) on a CFX Connect Real-Time PCR Detection System (Bio-Rad). Reactions were set up in a total volume of 10 µl, containing 5 µl SYBR Green Supermix, 0.3 µM of each forward and reverse primer, and 2 µl of 1:10 diluted cDNA template (corresponds to a final concentration of 1 ng/µl of total RNA equivalents). No-template controls were included in each run to exclude contamination and nonspecific amplification. Thermal cycling conditions comprised an initial polymerase activation step at 95°C for 15 min, followed by 35 amplification cycles with denaturation at 94°C for 15 s, primer-specific annealing at 60–64°C for 30 s, and extension at 72°C for 30 s. The following primer pairs were used: JAM-C #1, 5’-TCGGCCAGATGTGAGGAGCAG-3’, 5’-CAGGACGGCAAGGACCACCAG-3’; JAM-C #2, 5’- AGTGGTCCAGGAGTTTGAAAG-3’, 5’-CAACCACCTCACAGCGATAA-3’; JAM-C #3, 5’- CGACCGCAAAGAAATTGATGAG-3’, 5’-CAGAGTGCCTGTTTCAGAGTTT-3’; Occludin, 5’-CATGGTGGCCTTTTGTTTTATCG-3’, 5’-AGGGGGAGGAGGCATGTCTTGTG-3’; GAPDH, 5’-GCCATCACTGCCACCCAGAAGAC-3’, 5’-ATGCCAGTGAGCTTCCCGTTCAG-3’. Amplification specificity was verified by melt curve analysis. Quantification cycle (Cq) values were determined using the instrument’s software with automatic threshold settings. Relative gene expression was calculated using the 2⁻ΔΔCq method. Expression levels were normalized to the reference gene GAPDH, selected based on stable expression across experimental conditions. Data are presented as fold change relative to MDCKII WT cells.

### RNAseq experiments

RNAseq experiments were performed as described^75^. Preparation of cDNA libraries from total RNA and subsequent sequencing was performed using TruSeq Stranded mRNA LT sample prep kit (Illumina) and the Illumina platform. cDNA fragments obtained from RNAseq were mapped to the GCF_000002285.3 reference genome. For normalization of gene expression for each gene after mapping, trimmed mean of M-values normalization was performed using edgeR library. Subsequently, Fragments Per Kilobase of transcript per Million mapped reads (FPKM) and Transcripts Per Million (TPM) were calculated. RNAseq experiments were performed by Macrogen (Macrogen Japan Corp.).

### MDCK II cyst assays

MDCK II cells were adjusted to a single-cell suspension with a concentration of 6 × 10^4^ cells/ml in serum-free, ice-cold DMEM. The cell suspension was mixed 1:1 with ice-cold Matrigel matrix and plated into glass bottom cell culture slides (Greiner #543078). The gels were allowed to polymerize for 20 min at 37 °C in a humidified incubator with 5% CO_2_. After polymerization, 200 µl DMEM supplemented with 10% FCS was carefully added to the top of the gels and exchanged every two days. After four to six days MDCK cysts were fixed with 4% PFA for 1 h at RT. After fixation, cells were washed twice with PBS, 3% BSA and permeabilized in PBS, 0.1% Triton X-100 for 20 min at RT. Blocking was performed in PBS, 5% BSA for 1 h at RT. Antibody incubations were performed in PBS, 1% BSA, 0.05% Triton X-100. Primary antibodies were incubated overnight at 4°C, secondary antibodies were incubated for 2 to 3 h at RT. DNA was stained by incubating cysts in PBS, DAPI (1:400), 1% BSA, 0.05% Triton-X100. After three washes with PBS, 1% BSA, 0.05% Triton X-100, (10 min at RT each wash) and one wash with PBS, cysts were mounted by adding two drops of mounting medium (Ibidi #50001) per well. Cysts were analyzed using a confocal microscope as specified in the Immunocytochemistry and Immunofluorescence Microscopy section.

### Statistics and reproducibility

Results are expressed either as arithmetic means ± SD as indicated. To test the normality of data sample distributions, the D’Agostino-Pearson normality test was used. Data were statistically compared using unpaired, two-tailed Student’s *t*-test, or probed for being statistically different from a fixed value using One sample *t*-test. In cases of non-normal distrubution of data points, Kruskal-Wallis tests or Mann-Whitney-U tests followed by a Dunn’s multiple momparisons test were used. Statistical analyses were performed using GraphPad Prism version 6 (GraphPad Software, San Diego, CA). P-values are indicated as follows: *P < 0.05, **P < 0.01, ***P < 0.001 and ****P < 0.0001. For each statistical analysis data derived from at least three independent experiments were used. Experiments were considered independent when they reflected biological replicates acc. to Pollard et al.^76^. Sample sizes are indicated for all experiments in the respective figure legends.

## Supplementary Information

Below is the link to the electronic supplementary material.

Supplementary Material 1

Supplementary Material 2

## Data Availability

All data generated or analysed during this study are included in this published article and its supplementary information files.

## Abbreviations

AA: amino acid
IF: immunofluorescence
JAM: Junctional Adhesion Molecule
CGN: Cingulin
LLPS: Liquid-Liquid Phase Separation
PBM: PDZ domain-binding motif
PDZ: Postsynaptic density 95 - Discs Large - Zonula occludens-1
ZO: Zonula occludens
