# Supplementary material for: A direct interaction of JAM-C with the tight junction scaffold protein ZO-2
Source: Sci Rep. 2026 Jun 10;16:18005. doi: 10.1038/s41598-026-56546-x (PMC13253849; doi:10.1038/s41598-026-56546-x)
Supplement: Supplementary file 1 — Supplementary Material 1 [file 41598_2026_56546_MOESM1_ESM.docx]

**Supplementary Material**

**A direct interaction of JAM-C with the tight junction scaffold protein ZO-2**

Annika Schulte^1^, Mariel F. Schwietzer^1^, Frauke Brinkmann^1^, Valentin Teuber^1^, Sandra Citi^2^, Mikio Furuse^3^, Michel Aurrand-Lions^4^, Klaus Ebnet^1,5,*^

^1^Institute-associated Research Group "Cell adhesion and cell polarity", Institute of Medical Biochemistry, ZMBE; University Münster, Münster, Germany;

^2^Department of Molecular and Cellular Biology, University of Geneva, 1205 Geneva, Switzerland;

^3^Division of Cell Structure, National Institute for Physiological Sciences, National Institute of Natural Sciences, Okazaki, Aichi, Japan;

^4^Institut Paoli-Calmettes, CRCM, Equipe Labellisée Ligue 2020, Team “Leuko/stromal interactions in normal and pathological haematopoiesis”, Aix Marseille University, CNRS, INSERM, Marseille, France;

^5^Cells-in-Motion Cluster of Excellence (EXC 1003 - CiM), University of Münster, Münster, Germany;

^*^Correspondence

Klaus Ebnet, PhD, Institute-associated Research Group "Cell adhesion and cell polarity”, Institute of Medical Biochemistry, ZMBE, University of Münster, Von-Esmarch-Str. 56, D-48149 Münster, Germany

Tel.: +49-(0)251-8352127

FAX: +49-(0)251-8356748

e-mail: ebnetk@uni-muenster.de

Running title: JAM-C binds ZO-2


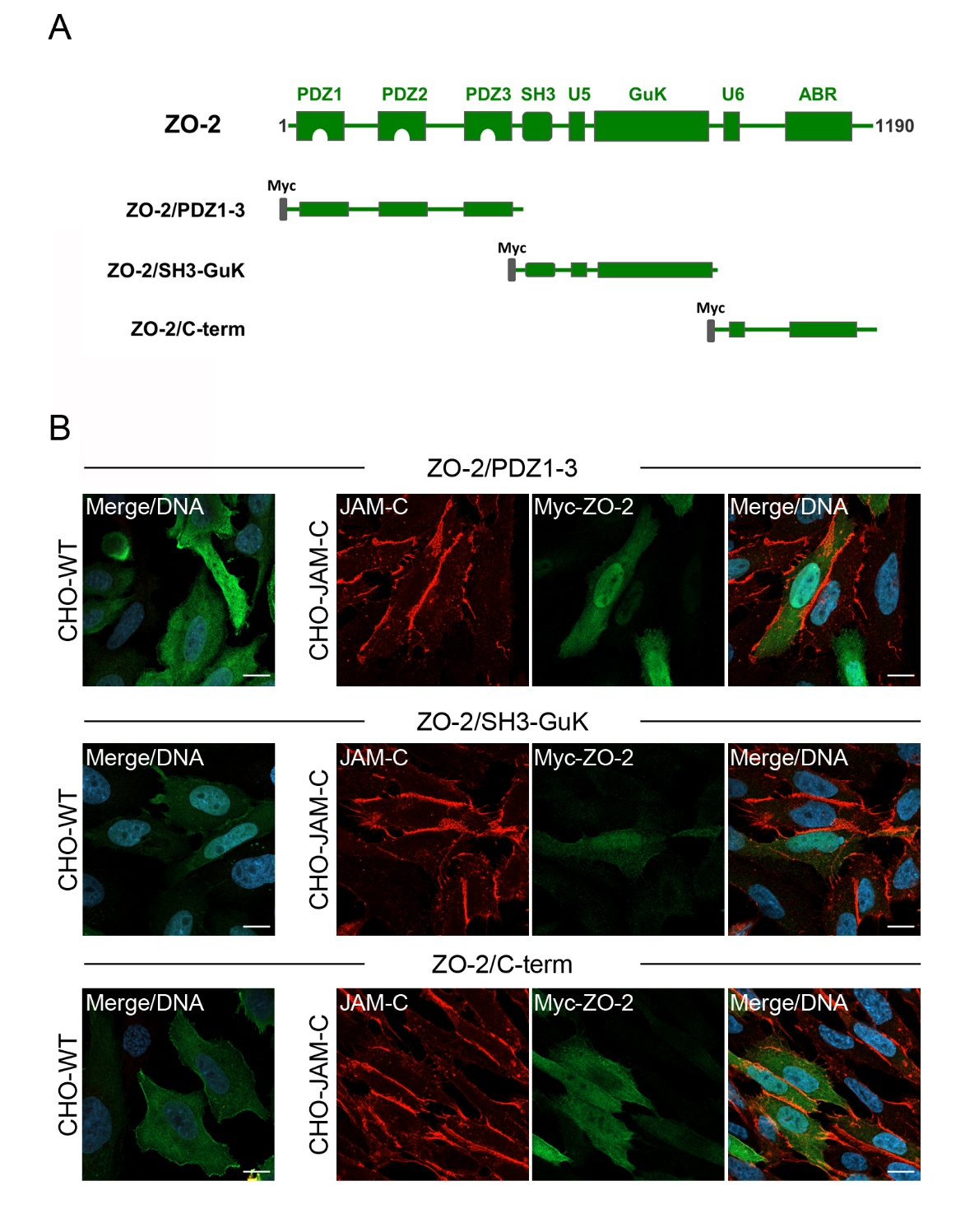


**Suppl. Fig. S1**: Recruitment of ZO-2 deletion constructs by JAM-C. (**A**) Cartoon of ZO-2 deletion constructs used in recruitment assays. (**B**) CHO-WT and CHO-JAM-C cells were transfected with Myc-tagged ZO-2 constructs shown in panel (A) and stained with antibodies against JAM-C (red) and against the Myc tag (green). Note that none of the three constructs is enriched at JAM-C-positive cell junctions. Data is representative of N = three independent experiments. Scale bars: 10 µm.


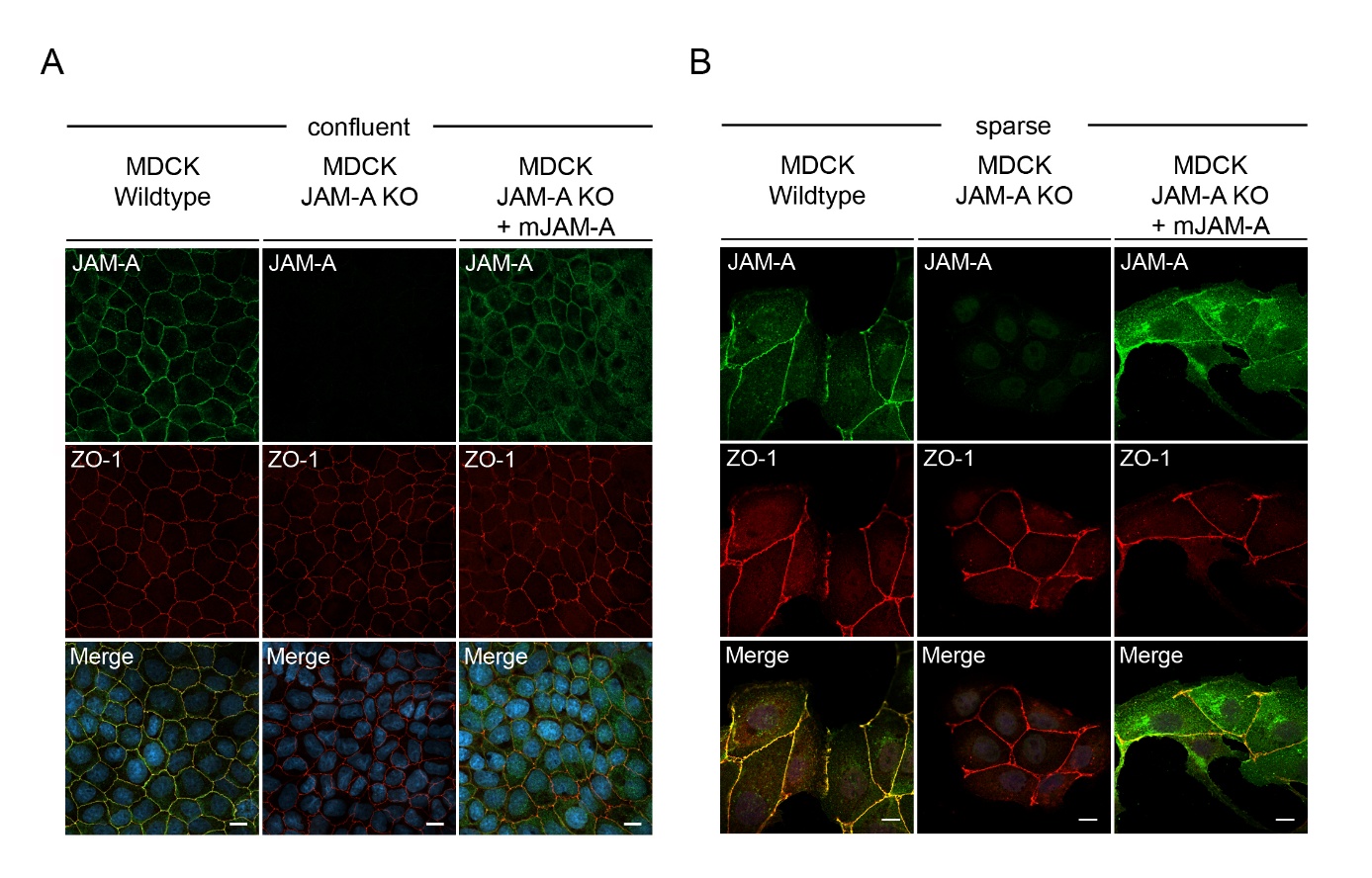


**Suppl. Fig. S2**: Expression of JAM-A in JAM-A KO MDCKII cells. (**A**) Wildtype MDCKII cells, JAM-A knockout MDCKII cells or JAM-A knockout MDCKII cells ectopically expressing mJAM-A were grown to confluency and stained with antibodies against JAM-A and ZO-1. Scale bars: 10 µm. (**B**) Wildtype MDCKII cells, JAM-A knockout MDCKII cells or JAM-A knockout MDCK II cells ectopically expressing mJAM-A were grown under sparse conditions and stained with antibodies against JAM-A and ZO-1. Scale bars: 10 µm.

**
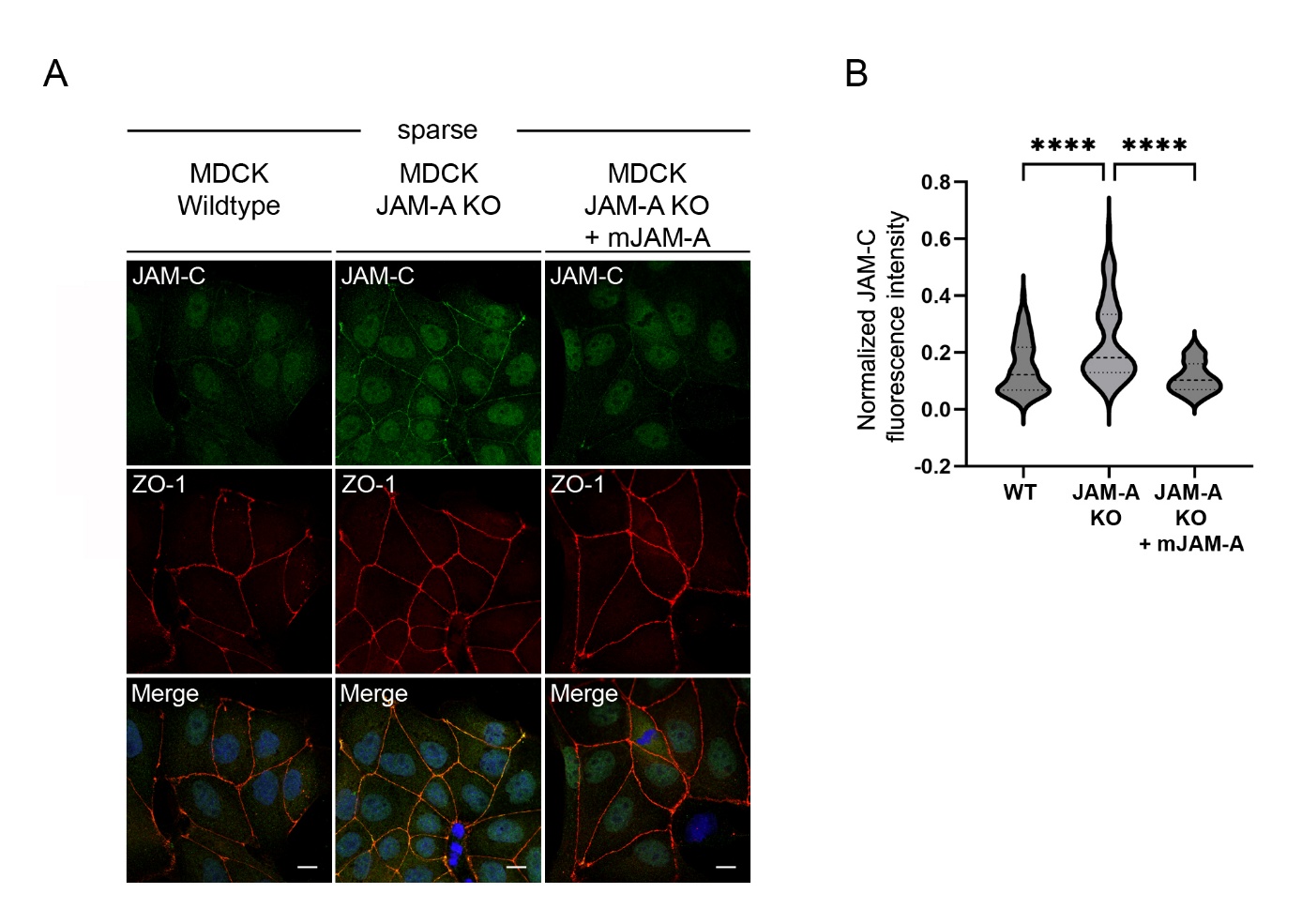
**

**Suppl. Fig. S3**: Localization of JAM-C in JAM-A KO MDCKII cells grown at sparse conditions. (**A**) Wildtype MDCKII cells, JAM-A knockout MDCKII cells or JAM-A knockout MDCK II cells ectopically expressing mJAM-A were grown under sparse conditions and stained with antibodies against JAM-C and ZO-1. Scale bars: 10 µm. (**B**) Statistical analysis of JAM-C localization at cells-cell contacts in sparse cells. The data shows the ratios of JAM-C fluorescence cell-cell contacts to the ZO-1 fluorescence intensity and is depicted as normalized JAM-C fluorescence intensity. Statistical analysis was performed with Kruskal-Wallis Test for multiple comparisons. Data were obtained and data points were pooled from at least 11 randomly chosen fields of view (FOV) per experiment derived from N = 3 independent experiments (WT cells: 67 FOV; JAM-A KO cells: 70 FOV; JAM-A KO cells expressing mJAM-A: 71 FOV). Data is presented as Violin plot. Broken lines indicate mean values, dotted lines indicate first and third quartiles. ****p < 0.0001.


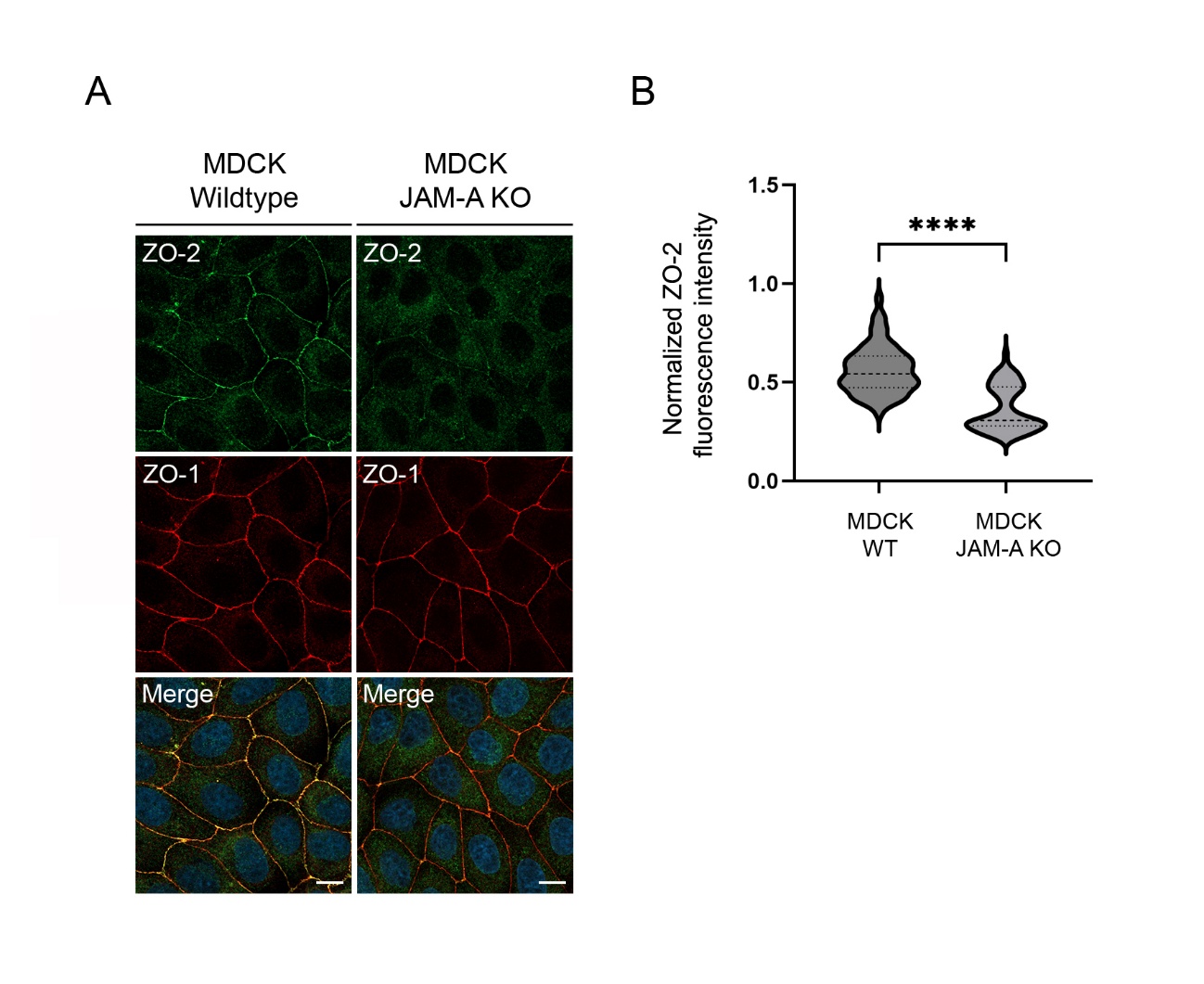


Suppl. Fig. S4: **Localization of ZO-2 in JAM-A KO MDCKII cells**. (**A**) Wildtype MDCKII cells and JAM-A KO MDCKII cells were stained with antibodies against ZO-2 and ZO-1. Scale bars: 10 µm. (**B**) Statistical analysis of ZO-2 localization at cell-cell contacts. The data show the ratios of ZO-2 fluorescence at cell–cell contacts to ZO-1 fluorescence intensity and are presented as normalized ZO-2 fluorescence intensity. Statistical analysis was performed with Mann-Whitney-U-Test. Data were obtained and data points were pooled from at least 20 randomly chosen fields of view (FOV) per experiment and are derived from N = 3 independent experiments (WT cells: 60 FOV; JAM-A KO cells: 60 FOV). Data are presented as Violin plot. Broken lines indicate mean values, dotted lines indicate first and third quartiles. ****p < 0.0001.


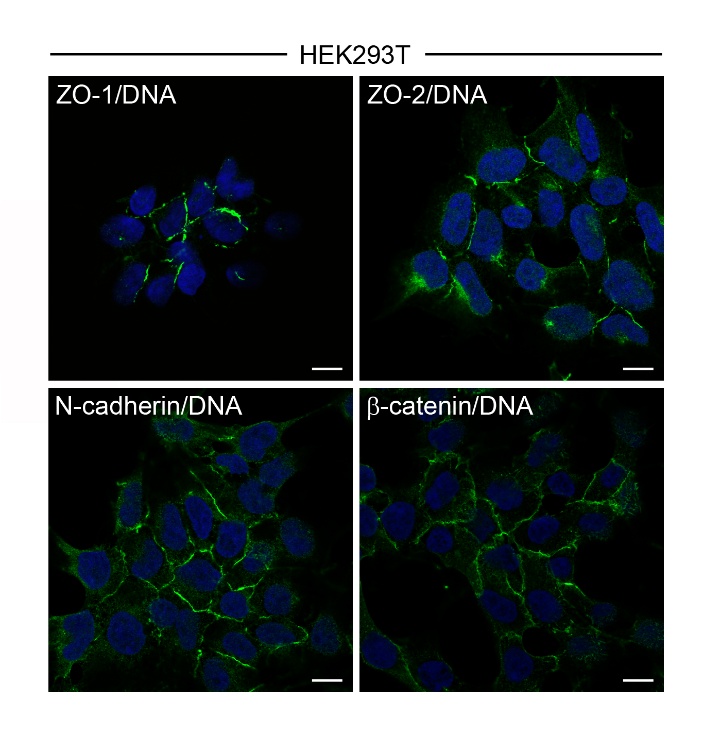


Suppl. Fig. S5: **Expression of ZO-1 and ZO-2 in HEK293T cells.** HEK293T cells were fixed and stained with antibodies against ZO-1, ZO-2, N-cadherin, and β-catenin as indicated. Note that both ZO-1 and ZO-2 are expressed in HEK293T cells and are localized at cell-cell contacts. Scale bars: 10 µm.

**Suppl. Table 1**: RNAseq analysis of TJ-associated integral membrane and scaffolding proteins known to interact with ZO-2 in MDCKII cells. Abbreviations: CAR, Coxsackie and adenovirus receptor; FPKM , Fragments Per Kilobase of transcript per Million mapped reads; TPM, Transcripts Per Million.

|  | **Gene ID** | **Gene Symbol** | **Read Count** | **FPKM** | **TPM** |
| --- | --- | --- | --- | --- | --- |
| **JAM-A** | 478974 | F11R | 32443 | 242,437134 | 477,124878 |
| **JAM-C** | 489271 | JAM3 | 168 | 1,093231 | 2,151518 |
| **CAR** | 403772 | CXADR | 13450 | 61,777306 | 121,579926 |
| **Occludin** | 403844 | OCLN | 3268 | 38,728783 | 76,219612 |
| **Claudin 1** | 608207 | CLDN1 | 17768 | 128,430878 | 252,756516 |
| **Claudin 2** | 403649 | CLDN2 | 44368 | 357,589386 | 703,748596 |
| **ZO-1** | 403752 | TJP1 | 6307 | 28,15999 | 55,419861 |
| **ZO-2** | 403854 | TJP2 | 3231 | 16,727978 | 32,921253 |
